# Religiosity, Spirituality and Mental Health: Meta-analysis of Studies from the German-Speaking Area

**DOI:** 10.1007/s10943-025-02406-3

**Published:** 2025-08-12

**Authors:** Christian Zwingmann

**Affiliations:** Protestant University of Applied Sciences RWL, Immanuel-Kant-Str. 18–20, 44803 Bochum, Germany

**Keywords:** Meta-analysis, Religiosity, Spirituality, Mental health, Germany

## Abstract

This meta-analysis of the relationship between religiosity/spirituality (R/S) and mental health includes 39 studies from the German-speaking area conducted between 2016 and 2022/23. The weighted average correlation is 0.083 (95% CI [0.055; 0.111]), which means that greater R/S is significantly associated with better mental health, but only to a small extent. The overall effect is only slightly larger than that found in a previous meta-analysis of studies from German-speaking countries (0.06 when excluding negative forms of R/S) and can be considered rather minimal compared to findings from meta-analyses that mainly include studies from the USA. The results are moderated to a minor extent by whether religiosity (R) or spirituality (S) is measured in the primary studies: The average correlation is higher for S and mixed scales than for R scales. Thus, in the more secularized German-speaking countries, it is more the non-institutionalized, conceptually and semantically open forms of transcendental meaning-making that are associated with mental health.

**Supplementary Information:**

The online version contains supplementary material available at 10.1007/s10943-025-02406-3.

## Introduction

Numerous meta-analyses show—for different settings (e.g., time periods, populations, outcomes)—that religiosity/spirituality (R/S) and mental health are positively correlated on average to a low to moderate extent: Lefevor et al. (2021) provide a tabular review of meta-analyses of the relationship between R/S and health for the period 1983 to 2019, 14 of which relate to mental health. Some more recent meta-analyses (Abdel-Khalek et al., 2019; Forouhari et al., 2019; Garssen et al., 2021; Je-se-rich et al., 2023; Yaden et al., 2022) are not yet included in the review by Lefevor et al. (2021). When these are also taken into account, the 19 available meta-analyses of the relationship between R/S and mental health yield overall effect sizes between 0.03 (Hodapp & Zwingmann, 2019) and 0.34 (Sawatzky et al., 2005), with both the mean and median being 0.14.

The meta-analysis with the smallest average effect size (Hodapp & Zwingmann, 2019; see also Zwingmann & Hodapp, 2018) refers to the German-speaking area (Germany, Austria, German-speaking Switzerland). This meta-analysis includes 67 primary studies published between 1990 and 2015/16. In addition to the minor overall effect, a comparatively strong negative mean correlation of – .20 was found between negative forms of R/S (negative religious coping, negative image of God or negative relationship with God, extrinsic religiosity) and mental health. Excluding negative forms of R/S from the analysis resulted in a slightly higher mean effect size of .06 (Hodapp & Zwingmann, 2019, p. 1989). However, this is still at the lower end of meta-analytically determined correlations to date.

For the overall low correlation between R/S and mental health in German-speaking countries found by Hodapp and Zwingmann (2019), the following two-part explanation is plausible. First, since most studies on the relationship between R/S and mental health have been conducted in the USA, this cultural area, with its specifically high level of religiosity (Bertelsmann Stiftung, 2008), is also overrepresented in the meta-analyses. The German-speaking area can be considered highly secularized by international standards (Bertelsmann Stiftung, 2022). The low average effect size found in the meta-analysis by Hodapp and Zwingmann (2019) may therefore indicate that the relationship between religiosity and mental health is weaker in a more secularized population.

Second, this explanation is also plausible because the R/S questionnaires available in German-speaking countries in the reference period 1990 to 2015/16 mostly operationalized religiosity, and instruments for measuring spirituality were still in their infancy (Klein et al., 2012; Zwingmann & Klein, 2012). However, as the importance of religion in society declines, people refer less and less to an institutionalized system of transcendental meaning-making (“religiosity”). This does not necessarily mean that they abandon transcendental attributions of meaning. Instead, they may prefer non-institutionalized, conceptually and semantically open forms of transcendental meaning-making (“spirituality”). Thus, in the more secularized German-speaking world, the relationship between R/S and mental health may be underestimated if spirituality is not adequately assessed.

In the meantime, several validated instruments for assessing spirituality are now available in German-speaking countries, and other questionnaires record mixed forms of R/S (Klein & Bethe, 2012; Zwingmann et al., in press). A new meta-analysis is therefore useful. In order to separate the correlations for religiosity and spirituality, the questionnaire scales used in the primary studies must be classified according to whether—or to what extent—they assess religious or spiritual transcendence. According to this understanding, both religiosity and spirituality refer, explicitly or at least implicitly, to some kind of “superhuman reality” beyond the meanings and experiences of an exclusively materially understood world. This also means that instruments that attempt to measure R/S without reference to transcendence must be excluded. This in turn helps to avoid artificially inflated correlations between R/S and mental health due to scale contamination (Garssen et al., 2016; Koenig & Carey, 2024). Although instruments without reference to transcendence are not necessarily flawed, Zwingmann et al.’s (in press) semantic analysis shows that they often reflect critical constructs such as positive experiences, a sense of peace, life motivation, growth and optimism about the future.

The new meta-analysis aims to update the meta-analytic findings on the relationship between R/S and mental health (Hodapp & Zwingmann, 2019) from the German-speaking area. For studies from the years 2016 to 2022/23, it will be examined whether the reported peculiarities for German-speaking countries persist in the face of increasing secularization, but also in the face of increased research efforts to further develop R/S measurement instruments. In particular, we are interested in whether the studies published between 2016 and 2022/23:

- Also show a very low overall correlation between R/S and mental health (which is lower than the correlations documented for the American area),
- Also show a clear negative correlation between negative forms of R/S and mental health (which is particularly strong compared to American studies),
- Show differences in the correlation between R/S and mental health depending on whether religiosity or spirituality was measured.

## Methods

The methodological approach follows the usual structure of meta-analytical studies: definition of inclusion and exclusion criteria, literature search, coding of the included primary studies, data analysis (overall effect size, heterogeneity tests, moderator analyses, assessment of publication bias). As an additional step before inclusion and coding of the studies, a classification of the measurement instruments used in the primary studies is carried out.

### Inclusion and Exclusion Criteria

The underlying inclusion and exclusion criteria are found in Table 1, with a brief explanation or justification where appropriate. The criteria are largely based on the previous meta-analysis by Hodapp and Zwingmann (2019).

**Table 1:** Inclusion and exclusion criteria

Inclusion Criteria	
I1	The study is published in German or English.
I2	The study is published as a journal article, monograph, book chapter or as a dissertation/habilitation.
I3	This is (partly) a quantitative empirical primary study, i.e., reviews, meta-analyses and study protocols are not taken into account.
I4	Except for minor deviations (≤ 10%), the sample comes from the German-speaking area (Germany, Austria, German-speaking Switzerland, South Tyrol).
I5	The study includes at least one quantitative measure of religiosity/spirituality.
I6	The study includes at least one quantitative measure of mental health.
I7	In the study, at least one correlation coefficient or partial association measure is specified as an effect size or can be calculated from the available information or data sets.
I8	The effect size is reported for cross-sectional data. This means that the measures of religiosity/spirituality and mental health on which the effect size is based must be available at the same point in time. They must not be averaged over several measurement points or represent differences.
I9	The sample size is specified and is *N* ≥ 30. Most statistics books recommend a sample size of *N* = 30, from which a normally distributed sample distribution of the mean values can be assumed.
Exclusion Criteria	
E1	The study is already included in the meta-analysis by Hodapp and Zwingmann (2019).
E2	The sample consists predominantly (> 50%) of refugees/migrants or people with a non-Christian background. The group of people with a non-Christian background does not include people who have either left the church or have no religious background but were socialized in the German-speaking cultural area. E2 was formulated so that the possible special circumstances of people with a migration background would not be disproportionately reflected in the overall effect size.
E3	The sample consists substantially (> 10%) of either a) children or adolescents, b) (future) religious leaders or members of religious orders, c) members of so-called new religious movements. E3 was formulated so that the possible special circumstances of the three mentioned groups of people are not disproportionately reflected in the overall effect size.
E4	The assessment of religiosity/spirituality is based solely on religious affiliation or measures of religious/spiritual needs. These measures cannot be considered adequate to operationalize religiosity/spirituality. Religious affiliation is too broad an indicator, and needs can be expressed without the presence of religiosity/spirituality.
E5	The assessment of religiosity/spirituality is based exclusively on measures that cannot be considered R/S measures.
E6	The mental health measures also assess physical health, or they assess only determinants of mental health (e.g., perceived stress, coping, resilience, sense of coherence).
E7	Effect sizes are reported selectively, e.g., only the significant correlations or predictors are reported.

### Literature Search, Screening and Eligibility Assessment

**Fig. 1:**
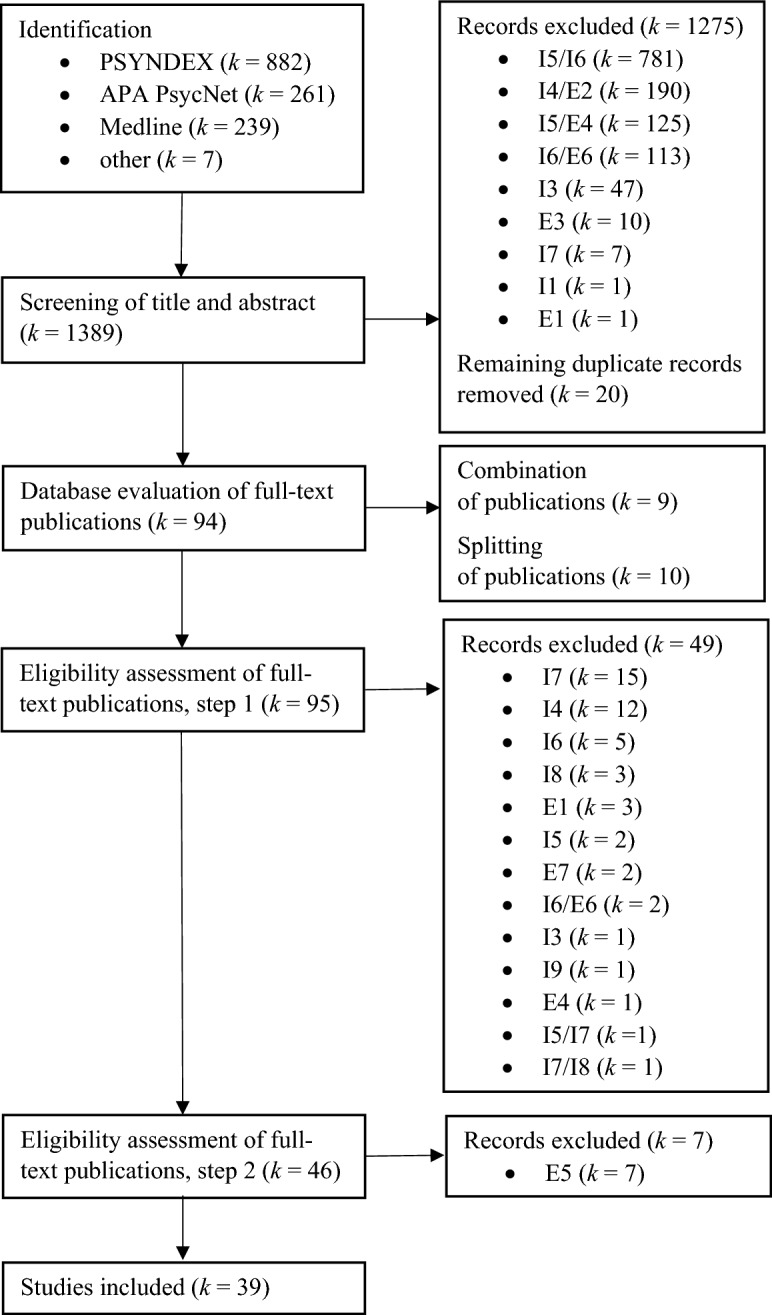
Flow diagram showing the progress of literature search, screening, and eligibility assessment (I = inclusion criterion, E = exclusion criterion)

The flow diagram shown in Fig. 1 provides an overview of the process.

Identification: For the literature search, the databases PSYNDEX (German), MEDLINE (via PubMed, English) and APA PsycNet (English) were systematically searched between March and May 2023 using a comprehensive search strategy. The respective Boolean search terms are based on the previous meta-analysis and are found in Appendix 1. The database search yielded 1382 hits; in addition, seven references from other sources were considered.

Screening: After title and abstract screening, 1275 references were clearly excluded according to the inclusion and exclusion criteria, followed by an additional 20 duplicates. The remaining 94 potentially relevant publications were obtained online or via inter-library loan.

Database evaluation of full-text publications: If an empirical study was published in more than one publication, and the presentations were therefore based on the same sample, these publications were combined (nine times). Conversely, if multiple samples were analyzed separately in a single publication, each of these samples was counted as a separate study (ten times). After this step, 95 studies were available.

Eligibility assessment of full-text publications: Eligibility assessment was performed in two steps. In the first step, all inclusion and exclusion criteria were checked, except for exclusion criterion E5, as this required a prior classification of the R/S instruments. In the first step, 49 studies were excluded (partly for different reasons). After the classification of the R/S instruments (see next section), criterion E5 was checked and seven more studies were excluded. Finally, 39 studies were included in the meta-analysis.

### Classification of R/S Measurement Instruments

The instruments used to measure R/S in all 46 studies remaining after the first step of the eligibility assessment were examined with regard to: (a) whether they measured religiosity (referring to an institutionalized system of transcendental meaning-making), spirituality (referring to non-institutionalized and conceptually/semantically open form of transcendental meaning-making), or a mixed form, and (b) which dominant contents they measured.

Regarding (a), the procedure proposed by Jeserich et al. (2023) was adapted: Each item of a questionnaire (sub)scale was coded on the basis of a semantic analysis as to whether it clearly contained religious (R) or spiritual (S) aspects, represented a mixed form (RS, RS/SR, SR/RS, SR), or did not address either religious or spiritual aspects (X). If a (sub)scale had more than 33% X codes, it was not considered to be an R/S instrument. A scale was coded as clearly R (or S) if ≥ 50% of the items were coded as R (or S) and at the same time < 50% of the items were coded as S (or R). If both R and S aspects were ≥ 50%, it was classified as one of the mixed forms (summarized for the analysis performed here).

Regarding b), the items of all (sub)scales that could be considered as R/S instruments were also coded in terms of content. The content coding is based on the aspects theoretically described by Zwingmann et al. (2011) and already considered in the meta-analysis by Hodapp and Zwingmann (2019): “salience/centrality” (1), various sub-aspects of centrality according to Huber and Huber (2012), namely “interest” (1.1), “ideology” (1.2), “experience” (1.3) and “practice” (1.4), as well as “consequences” (2), “positive religious/spiritual (r/s) coping/image/relationship” (3) and “negative r/s coping/image/relationship” (4). The two-digit numerical codes are intended to illustrate the hierarchical order in which 1.1, 1.2, 1.3 and 1.4 represent sub-aspects of “salience/centrality.” Again, each item was semantically analyzed and coded. A content aspect was considered to be scale dominant if it was represented in ≥ 50% of the items. A few (sub)scales had two dominant content aspects (1.2 + 1.3, 1.3 + 3).

For the classification of the instruments according to R/S and content, the interrater agreement was determined on the basis of two raters and three exemplary selected questionnaire scales with a total of 66 items. According to Altman (1991), interrater reliability can be considered as “good” with Cohen’s κ = 0.73 for the R/S classification and as “very good” with κ = 0.86 for the content classification.

The classification of the instruments according to R/S and content is shown in Appendix 2. A total of 91 (sub)scales (including single-item scales) were classified on the basis of 557 item codes. Regarding R/S classification, 30 (sub)scales can be classified as X (33.0%), 15 as R (16.5%), 18 as S (19.8%), and 28 as mixed forms (30.8%). The X scales were not coded further. The following contents dominate the remaining 61 (sub)scales: 15 times “salience/centrality” (24.6%), 12 times each “experience” and “positive r/s coping/image/relationship” (19.7% each), 10 times “practice” (16.4%), and 7 times “ideology” (11.5%). R/S and content coding are related: While R scales and mixed forms mainly contain the aspects “salience/centrality,” “positive r/s coping/image/relationship” and “practice,” the aspect “experience” is preferred for S scales. The aspect “ideology” appears mainly in mixed forms and S scales.

### Coding of Included Primary Studies

If available, the following information was extracted and coded for each primary study included: 1. author(s) and year of publication, 2. research area (Germany; Austria; German-speaking Switzerland; several), 3. year of survey (for multi-year surveys, the year with half the duration was coded), 4. sample with disease or in crisis situation (no; yes; mixed), 5. survey conducted during the COVID-19 pandemic (no; yes), 6. sample size, 7. proportion of women, 8. age of sample (*M*, *SD*), 9. denominational distribution (> 50% Catholic; > 50% Protestant; other), 10. indicators of mental health (only positive indicators; only negative indicators; both positive and negative indicators), 11. effect sizes, that is, reported or calculable correlation coefficients (Pearson’s correlation *r* or, in some cases, Spearman’s rank correlation *r*_s_) or, in four studies with regression coefficients, estimates according to the approach of Peterson and Brown (2005). The direction of the effect sizes was set uniformly so that positive values indicate that R/S is associated with better mental health. 12. R/S classification of the R/S instrument(s) used, 13. content classification of the R/S instrument(s) used.

The interrater reliability, determined for each category of the coding scheme on the basis of two raters and ten randomly selected primary studies, can be considered “good” to “very good” according to Altman (1991) (Cohen’s κ ≥ 0.63 with a median of κ = 0.89). The complete coding of all studies is shown in Table 2.

**Table 2:** Descriptions of the 39 studies included in the meta-analysis

No	Reference	Research area^a^	Year of survey	Sample type^b^	COVID-19^c^	*N*	Women [%]	Age: *M* (*SD*)	Denomina-tion^d^	Mental Health^e^	No. ES	Mean ES [95% CI]	R/S^f^	Content^g^
1	Aderhold et al. (2019), study 2	G	2015	Yes	No	251	52.6	59.1 (13.7)	Other	Both	3	0.027 [− 0.10, 0.15]	Mixed	3
2	Alewell et al. (2022)	G	2020	No	Yes	2551	46.1	44.1 (11.4)	N/A	Pos	2	0.060 [0.02, 0.10]	R, Mixed	1, 2
3	Budimir et al. (2021)	A	2020	No	Yes	1005	52.7	N/A (N/A)	N/A	Both	4	0.080 [0.02, 0.14]	Mixed	3
4	Burri et al. (2017)	S	N/A	Yes	No	43	76.7	51.8 (10.8)	N/A	Neg	2	0.071 [− 0.44, 0.18]	R	3
5	Butz et al. (2017)	G	2010	No	No	2319	50.1	49.7 (17.2)	N/A	Pos	2	0.155 [0.12, 0.20]	R	1, 1.4
6	Cwik and Büssing (2019)	G	N/A	Yes	No	201	65.7	37.4 (12.4)	N/A	Pos	3	− 0.056 [− 0.20, 0.08]	R, S, Mixed	1.4
7	Edinger-Schons (2020), study 1	G	2013	No	No	7066	72.4	38.0 (N/A)	N/A	Pos	1	0.183 [0.16, 0.21]	S	1.2
8	Edinger-Schons (2020), study 2	G	N/A	No	No	48,111	N/A	N/A (N/A)	Other	Pos	1	0.275 [0.27, 0.29]	S	1.2
9	Gebauer et al. (2017), study 1, Germany	G	2005	No	No	80,115	57.4	28.2 (N/A)	N/A	Pos	1	0.060 [0.05, 0.07]	R	1
10	Gebauer et al. (2017), study 1, Austria	A	2005	No	No	9070	59.7	26.9 (N/A)	N/A	Pos	1	0.070 [0.05, 0.09]	R	1
11	Gebauer et al. (2017), study 1, Switzerland	S	2005	No	No	13,278	56.1	29.1 (N/A)	N/A	Pos	1	0.070 [0.05, 0.09]	R	1
12	Hampel et al. (2019)	G	2013	No	No	2508	51.2	48.3 (19.1)	Other	Neg	4	− 0.065 [− 0.10, − 0.03]	R, S, Mixed	1, 1.1, 1.4
13	Hiebler-Ragger et al. (2016)	A	N/A	No	No	481	76.0	23.0 (2.9)	Cath	Neg	4	0.133 [0.04, 0.22]	Mixed	1
14	Höfer et al. (2020), sample 1	G	N/A	No	No	1073	55.0	45.0 (14.4)	N/A	Pos	1	0.220 [0.16, 0.28]	Mixed	1
15	Höfer et al. (2020), sample 2	A	N/A	No	No	685	65.3	26.0 (6.5)	N/A	Pos	2	0.095 [0.02, 0.17]	Mixed	1
16	Huber et al. (2020), sample 1	A	2018	No	No	584	63.4	20.8 (2.5)	N/A	Pos	1	0.200 [0.12, 0.28]	Mixed	1
17	Huber et al. (2020), sample 2	A	2018	No	No	274	62.0	34.2 (8.1)	N/A	Pos	1	0.010 [− 0.11, 0.13]	Mixed	1
18	Karwetzky et al. (2022)	G	2018	No	No	1597	62.9	N/A (N/A)	N/A	Pos	2	0.075 [0.03, 0.12]	Mixed	1
19	Klein and Bethe (2021), sample 1a	G	2012	No	No	317	61.2	29.7 (13.3)	Prot	Pos	1	− 0.060 [− 0.17, 0.05]	R	3
20	Klein and Bethe (2021), sample 2	G	2009	No	No	957	56.6	37.3 (13.5)	Other	Pos	1	0.040 [− 0.02, .010]	R	3
21	Klein and Bethe (2021), sample 3	G	2011	No	No	773	56.8	43.1 (14.0)	Other	Pos	4	0.205 [.013, 0.27]	S	13
22	Klein and Bethe (2021), sample 4; Klein et al. (2016)	G	2015	No	No	919	51.0	43.3 (14.3)	Other	Pos	7	0.082 [0.02, 0.15]	S, Mixed	1.2 + 1.3, 1.3, 3
23	Klein and Bethe (2021), sample 5	G	2005	No	No	146	73.4	23.2 (2.8)	Other	Pos	3	0.130 [− 0.03, 0.29]	S, Mixed	1.2 + 1.3, 3
24	Knorr et al. (2023), study 1	A	N/A	No	N/A	724	74.4	23.6 (4.1)	Cath	Neg	16	0.030 [− 0.04, 0.10]	R, S, Mixed	1.2, 1.3, 3
25	Kralovec et al. (2018); Plöderl et al. (2020)	A	2012	Yes	No	753	53.0	38.8 (N/A)	Cath	Neg	21	0.115 [0.04, 0.19]	R, S, Mixed	1, 1.1, 1.2, 1.3, 1.4
26	Peng-Keller et al. (2021)	S	2019	Yes	No	219	74.0	51.7 (15.0)	Other	Both	2	0.060 [− 0.07, 0.19]	S	1.3
27	Proyer and Laub (2017), sample 1	Sev	N/A	No	No	479	76.0	46.2 (12.2)	N/A	Pos	4	0.146 [0.06, 0.24]	S, Mixed	1, 1.2, 1.3
28	Reissmann et al. (2021)	G	2018	No	No	1863	63.7	85.1 (4.2)	N/A	Pos	14	0.183 [0.13, 0.22]	S, Mixed	1, 1.2, 1.3, 1.4, 1.3 + 3
29	Rojas et al. (2022)	G	N/A	Yes	N/A	106	36.8	29.8 (10.9)	N/A	Both	3	0.122 [− 0.07, 0.32]	Mixed	3
30	Schlechter et al. (2021), German residents	G	2018	No	No	205	68.3	26.3 (9.7)	N/A	Neg	1	0.050 [− 0.09, 0.19]	Mixed	3
31	Schmuck et al. (2021)	G	2020	Yes	Yes	4324	73.8	N/A (N/A)	N/A	Neg	2	0.060 [0.03, 0.09]	Mixed	3
32	Sorokowski et al. (2017), Germany	G	2013	No	No	101	58.4	47.7 (12.5)	Other	Pos	2	0.045 [− 0.15, 0.24]	R	1
33	Sorokowski et al. (2017), Switzerland	S	2013	No	No	172	39.5	49.4 (12.4)	Other	Pos	2	− 0.055 [− 0.21, 0.10]	R	1
34	Spitzenstätter and Schnell (2020)	Sev	2020	No	Yes	202	74.3	25.8 (6.6)	Cath	Neg	2	− 0.085 [− 0.22, 0.05]	Mixed	1, 1.2
35	Stavrova et al. (2016), study 2, Germany	G	2010	No	No	2903	N/A	N/A (N/A)	N/A	Pos	1	0.060 [0.02, 0.10]	R	1
36	Surall and Steppacher (2020)	G	N/A	No	No	652	62.0	49.0 (N/A)	Other	Pos	1	0.110 [0.03, 0.19]	R	1.2
37	Teismann et al. (2017)	G	N/A	Both	No	309	67.7	32.8 (N/A)	Other	Neg	1	0.168 [0.06, 0.28]	Mixed	3
38	Vuzic et al. (2022)	A	2020	No	Yes	306	72.5	22.2 (3.8)	N/A	Neg	4	0.061 [− 0.12, 0.11]	R, S	1.3, 3
39	Zacher and Rudolph (2021)	G	2020	No	Yes	979	40.2	44.5 (10.9)	N/A	Both	3	− 0.041 [− 0.10, 0.02]	Mixed	3

*N/A* No data available

^a^Research area: G = Germany, A = Austria, S = German-speaking Switzerland, Sev = Several

^b^Sample with disease or in crisis situation?

^c^Survey conducted during the COVID-19 pandemic?

^d^Denomination: Cath = Catholic > 50%, Prot = Protestant > 50%, Other = other distribution

^e^Indicators of Mental health: Pos = only positive indicators, Neg = only negative indicators, Both = both positive and negative indicators

^f^R/S classification of the instrument(s) used in the study: R = Religiosity scale, S = Spirituality scale, Mixed = mixed scale

^g^Content classification of the instrument(s) used in the study: 1 = salience/centrality in general, 1.1 = interest, 1.2 = ideology, 1.3 = experience, 1.4 = practice, 2 = consequences, 3 = positive religious/spiritual coping/image/relationship

### Data Analysis

The calculation of the weighted mean overall effect size *r* + was based on a random effects model with iterative approximate REML estimation, which is based on the assumption that true effect sizes are not constant across primary studies, but vary (Borenstein et al., 2009). Although studies with larger samples are given greater weight in the random effects model, the increase is not linear, so that the specific information from smaller studies is also adequately represented (Borenstein et al., 2009, Chapter 13). Each study was included in the calculation of the overall effect size with only one effect size. If a primary study provided multiple effect sizes, these were first aggregated to a mean study effect size (using Fisher’s *Z*-transformation).

The *Q* statistic and the *I*^2^ statistic were used to quantify the heterogeneity of the primary studies (Borenstein et al., 2009, Chapter 16). Moderator analyses were then performed. Subgroups were created according to coded study and sociodemographic characteristics, and a weighted mean effect size *r* + was calculated for each subgroup using the random effects model. The *Q* statistic was used to quantify the heterogeneity of the subgroups. Similarly, moderator analyses were conducted according to the R/S and content classification of the instruments. These were dependent subgroups because it was possible that different R/S and/or content aspects were measured in the same study. If multiple effect sizes were available for an R/S or content aspect in a study, these were aggregated (using Fisher’s *Z*-transformation) before conducting the respective moderator analysis.

To assess whether the overall effect size was robust to publication bias, a funnel plot was generated (see, e.g., Bax et al., 2008). In this plot, the study size (as a standard error) is plotted on the ordinate and the study effect size (as a Fisher *Z*-value) is plotted on the abscissa. Studies with large sample sizes appear at the top of the graph, and studies with small sample sizes appear at the bottom. In the absence of publication bias, studies should be distributed symmetrically on either side of the mean effect size. As the funnel plot produced a partially asymmetric picture, the trim-and-fill method (see, e.g., Borenstein et al., 2009, pp. 286f.) was also applied. The asymmetric distribution is balanced by imputing the appropriate studies and recalculating the mean effect size.

All analyses were performed using SPSS for Windows, version 28. Some preliminary and supplementary calculations were performed using Excel 2016.

## Results

### Descriptive Characteristics

The meta-analysis included *k* = 39 independent primary studies (publication period: 2016–2023) with a total of *N* = 188,561 participants. From these studies, 131 effect sizes were extracted, resulting in an average of 3.36 effect sizes (*SD* = 4.28, *min* = 1, *max* = 21) per study.

Table 3 shows the following information: The primary studies were conducted between 2005 and 2020, spanning 16 years, with most studies (9 times, 23.1%) being conducted between 2011 and 2015. The majority of the studies are from Germany (24 times, 61.5%) and mainly include healthy and unstressed individuals (31 times, 79.5%). Only six studies (15.4%) were conducted during the COVID-19 pandemic. Most samples include between 251 and 1000 persons (16 times, 41.0%) or even more than 1000 persons (14 times, 35.9%) and contain more women (20 times, 51.3%). On average, the study participants are mostly younger than 30 years old (17 times, 43.6%) or between 30 and 60 years old (16 times, 41.0%), so they are relatively young. Only in about one tenth of the samples is more than half of the study population either Catholic (3 times, 7.7%) or Protestant (1 time, 2.6%). Most studies used only positive indicators to measure mental health (24 times, 61.5%, most often: life satisfaction, psychological well-being), but there are also a considerable number of studies that used only negative indicators (10 times, 25.6%, most often: depression, anxiety, psychological distress) or both positive and negative indicators (5 times, 12.8%).

**Table 3:** Study and sociodemographic characteristics of primary studies and moderator analyses: *Q* statistic, number of studies, and effect sizes *r* + of subgroups

	Number of studies	Effect sizes *r* + of subgroups [95% CI]
*Survey period:* Δ*r* + = 0.080 (*Q* = 4.99, *df* = 3, *p* = 0.17)		
Before 2011	7	0.077** [0.050, 0.105]
2011–2015	9	0.061 [− 0.012, 0.133]
2016–2019	6	0.108** [0.045, 0.170]
2020	6	0.028 [− 0.017, 0.073]
No data available	11	0.126** [0.065, 0.188]
*Research area:* Δ*r* + = 0.054 (*Q* = 1.87, *df* = 3, *p* = 0.60)		
Germany	24	0.091** [0.052, 0.131]
Austria	9	0.085** [0.050, 0.121]
German-speaking Switzerland	4	0.042 [− 0.022, 0.106]
Several	2	0.037 [− 0.190, 0.264]
*Sample with disease or in crisis situation:* Δ*r* + = 0.108 (*Q* = 4.24, *df* = 2, *p* = 0.12)		
No	31	0.086** [0.054, 0.118]
Yes	7	0.062** [0.036, 0.087]
Mixed	1	0.170** [0.058, 0.282]
*Survey during the COVID-19 pandemic:* Δ*r* + = 0.069 (*Q* = 5.86, *df* = 1, *p* = 0.02)		
No	31	0.097** [0.065, 0.129]
Yes	6	0.028 [− 0.017, 0.073]
No data available	2	0.042 [− 0.027, 0.110]
*Sample size:* Δ*r* + = 0.237 (*Q* = 7.55, *df* = 3, *p* = 0.06)		
< 100	1	− 0.131 [− 0.441, 0.179]
101–250	8	0.017 [− 0.040, 0.074]
251–1000	16	0.081** [0.042, 0.121]
> 1000	14	0.106** [0.060, 0.153]
*Proportion of women:* Δ*r* + = 0.059 (*Q* = 0.45, *df* = 2, *p* = 0.08)		
< 40%	2	0.023 [− 0.149, 0.195]
40–60%	15	0.076** [0.036, 0.117]
> 60%	20	0.082** [0.045, 0.120]
No data available	2	0.172 [− 0.046, 0.389]
*Mean age:* Δ*r* + = 0.100 (*Q* = 13.51, *df* = 2, *p* = 0.00)		
*M* < 30 years	17	0.075** [0.041, 0.110]
30 years < *M* < 60 years	16	0.073** [0.022, 0.124]
*M* > 60 years	1	0.175** [0.130, 0.220]
No data available	5	0.113* [0.026, 0.200]
*Denomination:* Δ*r* + = 0.151 (*Q* = 5.49, *df* = 2, *p* = 0.06)		
Catholic > 50%	3	0.033 [− 0.071, 0.137]
Protestant > 50%	1	− 0.060 [− 0.171, 0.051]
Other	12	0.091** [0.027, 0.156]
No data available	23	0.091** [0.061, 0.120]
*Indicator of mental health:* Δ*r* + = 0.106 (*Q* = 5,96, *df* = 2, *p* = 0.05)		
Only positive indicators	24	0.105** [0.071, 0.140]
Only negative indicators	10	0.041 [− 0.014, 0.095]
Both positive and negative indicators	5	0.036 [− 0.027, 0.099]

Δ*r* + : difference between the highest and the lowest effect size of subgroups for each characteristic (without the category “No data available”)

*p < 0.05, **p < 0.01

When aggregating the sociodemographic data across all primary studies (if available for the extracted effect sizes), the following overall distributions emerge: The gender of the study participants is available in 37 studies (94.8%), in which a total of 61.1% of the study participants are female. The mean age is available in 34 studies (87.2%) and amounts to a weighted mean of 31.4 years. The distribution of religious/denominational affiliation is available in only 16 studies (41.0%). Of these, 26.9% are Catholic, 34.6% are Protestant, 28.2% have no religious affiliation, and 12.5% have another religion.

Most studies use only scales that are a mixture of R and S (14 times, 35.9%), followed by studies that use only R scales (11 times, 28.2%). Just a few studies use only S scales (4 times, 10.3%). The remaining 10 studies (25.6%) include instruments from several of these three groups, so that a total of 23 studies (59.0%) use mixed instruments, 17 studies (43.6%) use R scales and 13 studies (33.3%) use S scales. Regarding the content classification of the R/S measures, most studies (28 times, 71.8%) use scales with only one dominant content aspect, most frequently “salience/centrality” (12 times, 30.8%) and “positive r/s coping/image/relationship” (10 times, 25.6%). The remaining 11 studies (28.2%) use R/S measures with different foci simultaneously. The content aspect “negative r/s coping/image/relationship” does not appear in any study.

### Mean Effect Size

**Fig. 2:**
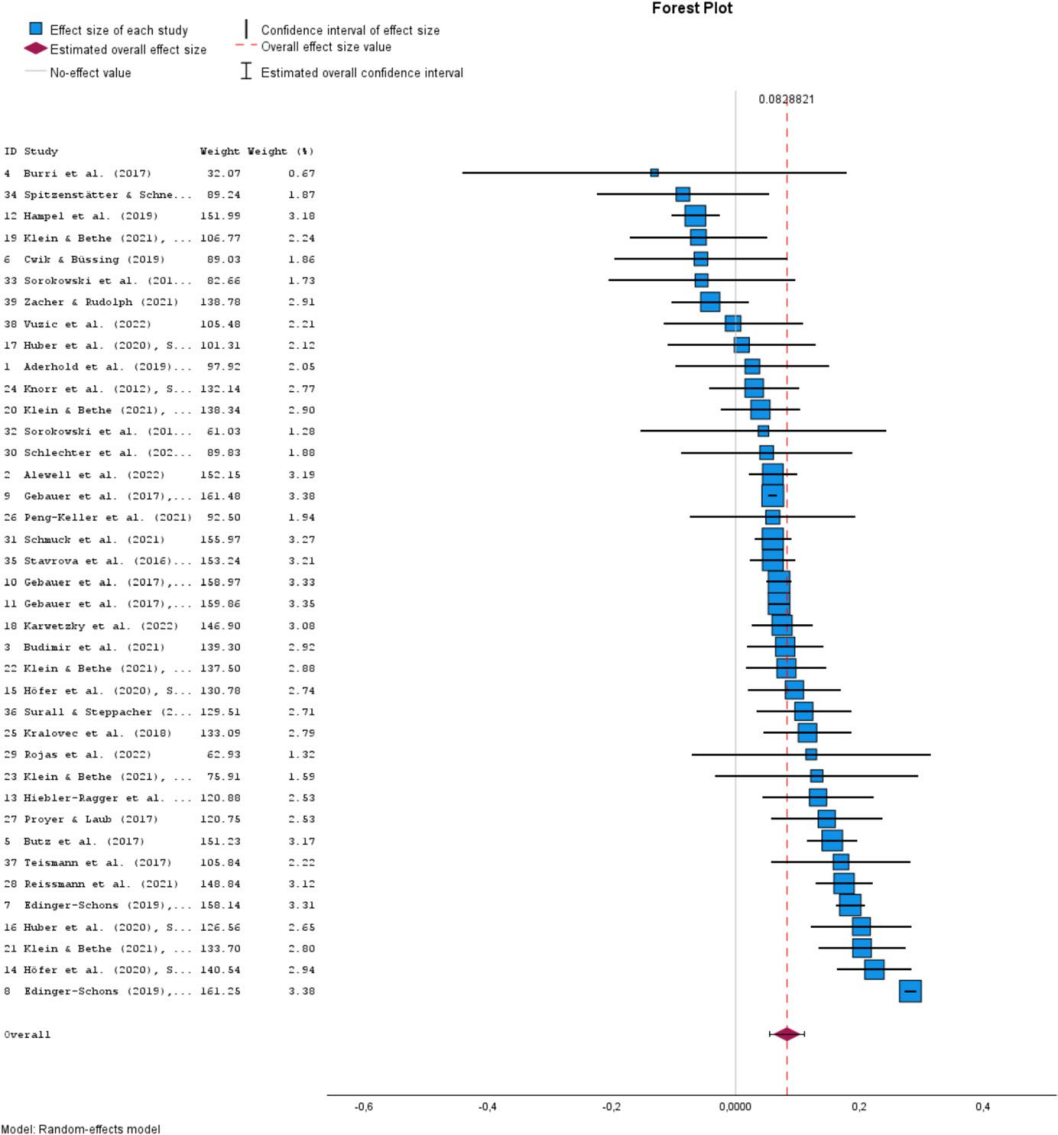
Forest plot for the 39 primary studies that were included in the meta-analysis. For each primary study, the mean study effect size *r*—ordered by size—and its 95% CI are shown. The overall mean effect size *r* + with its 95% CI is shown at the bottom

Of the 39 study-specific mean effect sizes *r*, 7 (17.9%) are negative and 32 (82.1%) are positive, ranging from − 0.13 to 0.28. The overall mean effect size weighted according to the random effects model is *r* +  = 0.083 (*z* = 5.727, *p* < 0.001, 95% CI [0.055; 0.111]). Figure 2 illustrates these results as a forest plot. Note that the (percentage) weighting factors of all studies are also shown in Fig. 2. The largest primary study (Gebauer et al., 2017, study 1, Germany) contains 80,115 participants (see Table 2, No. 9), which is 42.5% of all participants included in the meta-analysis. However, as can be seen in Fig. 2, the percentage weighting factor of this study in estimating the overall effect size under the random effects model is only 3.38%. The smallest primary study (Burri et al., 2017) contains 43 participants (see Table 2, No. 4), which is 0.02% of all persons included in the meta-analysis. In contrast, the weighting factor of this study in the estimate of the overall effect size is 0.67%.

### Moderator Analyses

The heterogeneity between studies, already apparent in the forest plot, was quantitatively confirmed by heterogeneity tests: The *Q* statistic (*Q* = 1873.41, *df* = 38, *p* < 0.01) indicates that the included primary studies do not share a common effect size. The *I*^2^ statistic reaches a value of 96%, denoting that a large proportion of the observed variance is real variance in the true effects, which according to Higgins et al. (2003) is an indication of strong heterogeneity.

Moderator analyses were conducted to find possible explanations for the heterogeneity between studies. The results for coded study and sociodemographic characteristics are shown in Table 3. Except for “survey during the COVID-19 pandemic” and “mean age,” the *Q* statistic is not statistically significant. However, this result should not be interpreted too strictly because the significance depends largely on the subgroups to which the two largest primary studies (Edinger-Schons, 2020, study 2; Gebauer et al., 2017, study 1, Germany) are assigned. The mean effect sizes *r* + of the subgroups are mostly quite close to each other; the differences Δ*r* + between the highest and lowest subgroup effect sizes (excluding the “no data available” category) mostly range only from 0.054 to 0.108. The heterogeneity of the study effect sizes can therefore only be explained to a limited extent by the corresponding characteristics. Slightly higher Δ*r* + differences of 0.151 and 0.237 are found for the variables “denomination” and “sample size,” respectively.

Within these rather small differences, higher effect sizes are found for studies conducted between 2016 and 2019, for studies from Germany and Austria, for studies that included people who were not ill or distressed, for studies not conducted during the COVID-19 pandemic, for studies with large samples, for studies with a high proportion of women, for a study with older people, for studies with mixed denominational samples, and for studies with only positive indicators of mental health.

The moderator analyses, which were conducted according to the classification of the measures with regard to R/S and content, are presented in Table 4. The moderator analysis for R/S shows, with a very small difference Δ*r* +  = 0.031 and a nonsignificant *Q* statistic, that the average effect size increases from R scales (*r* +  = 0.056) to mixed scales (*r* +  = 0.083) and S scales (*r* +  = 0.087). The moderator analysis with regard to content aspects yields a higher difference Δ*r* +  = 0.109 and a significant *Q* statistic. However, if only those content categories are considered for which at least five effect sizes are present, the difference drops to Δ*r* +  = 0.077; the strongest effect sizes are then found for “ideology” (*r* +  = 0.127) and “experience” (*r* +  = 0.110), while smaller effect sizes are found for “salience/centrality” (*r* +  = 0.076), “practice” (*r* +  = 0.068), and especially for “positive r/s coping/image/relationship” (*r* +  = 0.050).

**Table 4:** Moderator analyses for R/S and content: *Q* statistic, number of studies, and effect sizes *r* + of subgroups

	Number of studies	Effect sizes *r* + of subgroups [95% CI]
*R/S classification of instruments:* Δ*r* + = 0.031 (*Q* = 1.59, *df* = 2, *p* = 0.45)		
Spirituality scales	13	0.087* [0.002, 0.171]
Mixed scales	23	0.083** [0.048, 0.117]
Religiosity scales	17	0.056** [0.027, 0.084]
*Content classification of instruments:* Δ*r* + = 0.109 (*Q* = 24.65, *df* = 8, *p* = 0.00)		
Ideology (1.2)	8	0.127** [0.042, 0.212]
Mixed: Experience + positive r/s coping/image/relationship (1.3 + 3)	1	0.110** [0.065, 0.155]
Experience (1.3)	8	0.110* [0.025, 0.196]
Mixed: Ideology + Experience (1.2 + 1.3)	2	0.098** [0.038, 0.158]
Consequences (2)	1	0.092** [0.053, 0.131]
Salience/centrality (1)	19	0.076** [0.040, 0.112]
Practice (1.4)	5	0.068 [− 0.039, 0.174]
Positive r/s coping/image/relationship (3)	14	0.050** [0.020, 0.079]
Interest (1.1)	2	0.000 [− 0.035, 0.035]
Negative r/s coping/image/relationship (4)	0	–

Δ*r* + : difference between the highest and the lowest effect size *r* + of subgroups for each classification. The subgroups are sorted in descending order by the magnitude of the effect size *r* +

*p < 0.05, **p < 0.01

### Assessment of Publication Bias

**Fig. 3:**
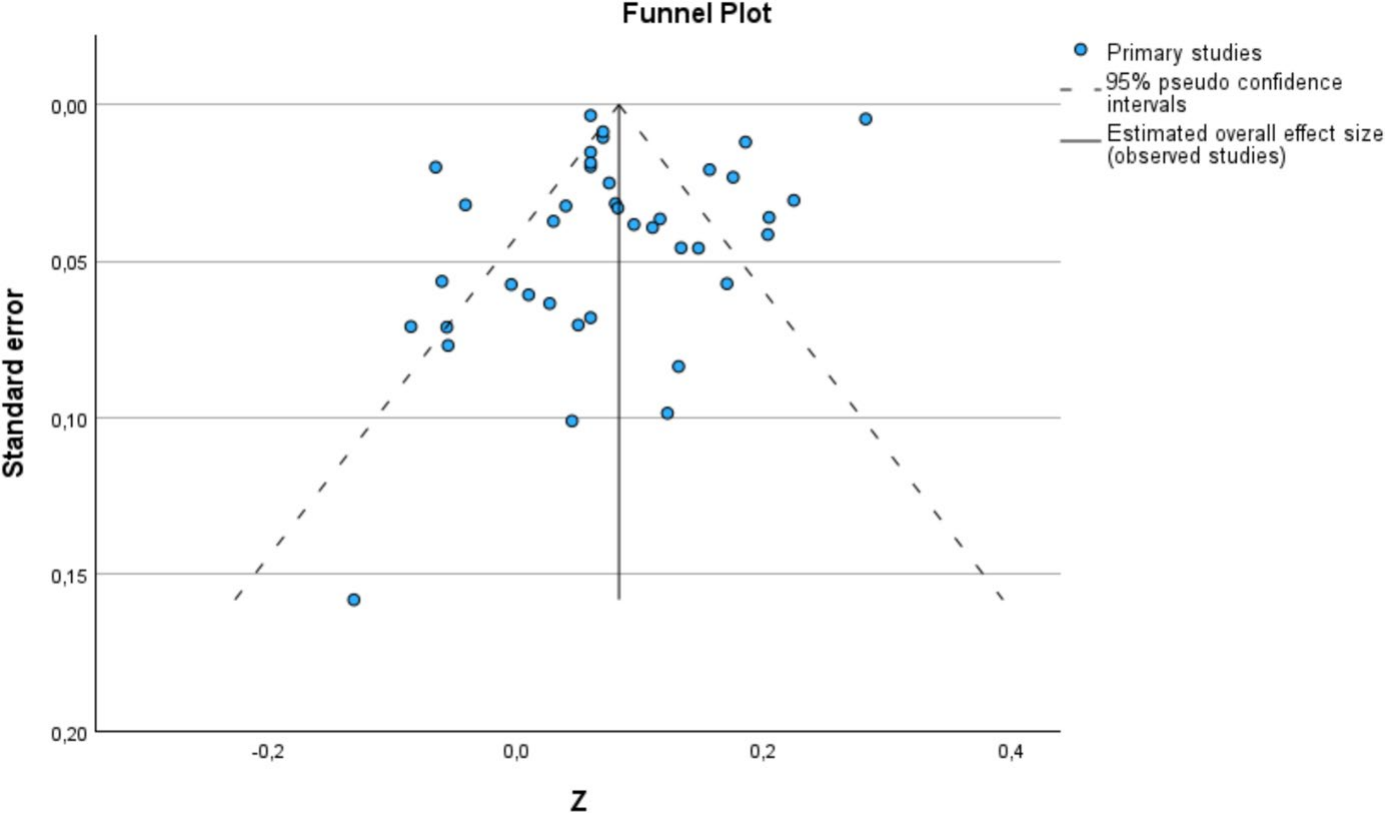
Funnel plot. The study size (as a standard error) is plotted on the ordinate, and the study effect size (as a Fisher *Z*-value) is plotted on the abscissa

**Fig. 4:**
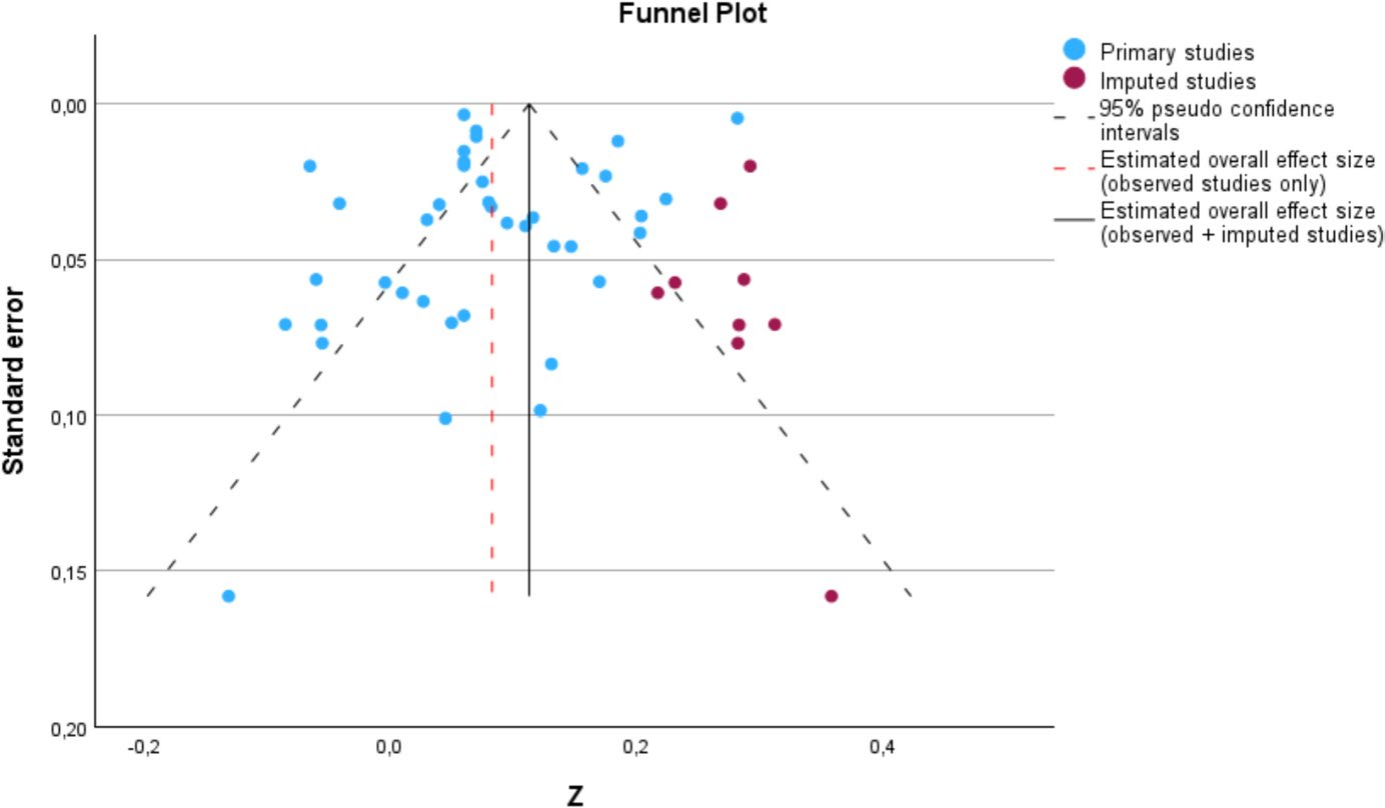
Funnel plot with imputed studies according to the trim-and-fill method

The funnel plot in Fig. 3 shows a fairly symmetrical picture overall. However, there are slightly fewer studies with low and medium standard errors on the right side of the funnel than on the left side of the mean effect size. The trim-and-fill method was used to impute the missing studies to compensate for the asymmetric distribution. Nine values were added to the right side of the graph. The recalculated mean effect size is *r* +  = 0.113 (95% CI [0.082; 0.144]). Figure 4 shows the funnel plot with the nine imputed studies.

One assumption about publication bias is that it mainly affects small studies, because studies with large samples tend to be published independently of their results. However, in the trim-and-fill procedure described above, it was mainly medium to large studies that were imputed. In addition, it can be seen that the mean effect size shifts to a slightly higher range when the imputed studies are included. This is contrary to the pattern that would be expected from publication bias if results with small or nonsignificant effect sizes were withheld. A plausible explanation for this is that in a substantial number of the primary studies included in the present meta-analysis, the relationship between R/S and mental health is not the focus of research interest. Rather, the effect size of interest here is only provided as a by-product, for example, when validating a measurement instrument or investigating some other substantive research question. It is therefore reasonable to assume that the overall effect size estimate is not significantly biased by publication bias.

## Discussion

The present meta-analysis includes a total of 39 primary studies for the seven-year period between 2016 and 2022/23, which averages about five studies per year. In the previous meta-analysis from German-speaking countries (Hodapp & Zwingmann, 2019, Table 2), an increase in relevant publications was observed from 2006, with an average of 4.1 publications per year until 2016. In the following years until 2022/23, this publication frequency showed another slight increase.

The current meta-analysis does not include a single study assessing negative forms of R/S, such as negative religious coping or negative feelings toward a transcendent being. Although some German studies have addressed “spiritual dryness” as a form of spiritual crisis (e.g., Baumann et al., 2019; Büssing et al., 2017), these studies focused on religious leaders and were excluded from this meta-analysis based on exclusion criterion E3. It should be noted for publications since 2016 that the relationship between negative forms of R/S and mental health has not been empirically examined in German-speaking samples from the general population. This is a remarkable finding, especially considering the substantial research on spiritual struggle and its relationship to mental health and distress in the United States (Exline et al., 2024; Pargament & Exline, 2022). The omission of negative forms of R/S in German-language research in general population samples may both result from and perpetuate the overestimation of R/S’s positive potential for mental health (Zwingmann et al., 2017). In fact, the previous meta-analysis from German-speaking countries revealed that negative forms of R/S had, on average, a relatively strong negative effect on mental health (*r* +  = − 0.20; Hodapp & Zwingmann, 2019, Table 3).

The weighted mean overall effect size calculated in the present meta-analysis across all 39 included primary studies is *r* +  = 0.083. Thus, at first glance, the present meta-analysis appears to show a stronger average association between R/S and mental health than the previous one (*r* +  = 0.03; Hodapp & Zwingmann, 2019). However, the current meta-analysis does not include studies that report (also) negative forms of R/S. If effect sizes in this regard are excluded from the previous meta-analysis, this results in a higher mean effect size of *r* +  = 0.06 (Hodapp & Zwingmann, 2019, p. 1989). This means that the current meta-analysis results in an overall only slightly higher estimate of the mean association between R/S and mental health than the previous one. However, compared to the findings of Hodapp and Zwingmann (2019, p. 1983), the range of study effect sizes was substantially reduced, namely from 0.81 [− 0.41; 0.40] to 0.41 [− 0.13; 0.28]. This may indicate a reduced methodological variability due to improved measurement instruments.

The overall effect size now determined, *r* +  = 0.083, represents a positive correlation between R/S and mental health that is significantly different from zero, but can at best be described as “small” according to Cohen (1988). Compared to the meta-analytically determined correlations in the USA (0.06 ≤ *r* +  ≤ 0.19; cf. the overview in Zwingmann, 2022, p. 327f.), it is in the lower range. In this respect, the present meta-analysis confirms that R/S and mental health are on average only slightly related in the highly secularized German-speaking area, and less so than has been documented for the American area.

Although the range of primary effect sizes in the present meta-analysis is smaller than in the previous meta-analysis from German-speaking countries, it can still be considered strong according to the heterogeneity tests performed. For this reason, numerous moderator analyses were performed. For this purpose, subgroups were formed according to various characteristics considered in the coding and subgroup-specific effect sizes were calculated. For study and sociodemographic characteristics, as in the previous meta-analysis, mostly small differences (0.054 ≤ Δ*r* +  ≤ 0.108) were found between the subgroups. In detail, the small differences already reported in the previous meta-analysis were confirmed for the variables “survey period,” “sample with disease or in crisis situation,” “proportion of women,” and “indicators of mental health” (cf. Hodapp & Zwingmann, 2019, Table 2). Contrary to the findings of the preceding meta-analysis, the current analysis also shows only limited differences in the subgroup effect sizes for the characteristics “research area” and “mean age.” However, the subgroup differences for “mean age” are significant, due to a deviating subgroup with only one study on older people. This may indicate that older people in German-speaking countries tend to use R/S for social support, hope and comfort. The subgroup differences for the newly coded characteristic “survey during the COVID-19 pandemic” are also small but significant. The particularly small effect size (*r* +  = 0.028) for studies conducted during the pandemic may indicate that there was a lack of social support in the religious sphere during periods of lockdown, with greatly reduced opportunities for face-to-face contact at church services and in parish work.

For the two characteristics “sample size” (Δ*r* +  = 0.234) and “denominational distribution” (Δ*r* +  = 0.143), the present meta-analysis shows slightly higher differences between the respective subgroups, but these are each also due to a deviating subgroup with only one study. Regarding the characteristic “sample size,” the effect size of the subgroups grows with increasing sample size. This can probably be explained methodologically, as higher correlations can only result if R/S and mental health vary substantially, which is more plausible with larger samples. With regard to the characteristic “denominational distribution,” it is hardly possible to draw any substantive conclusions, as there are only four studies in which Catholics or Protestants predominate. Most of the studies do not provide information on the denominational distribution.

As a further part of the moderator analyses, the effect sizes of the R/S measures were compared according to their classification by R/S and content. For the R/S classification, the correlation with mental health is higher for spirituality (*r* +  = 0.087) and mixed scales (*r* +  = 0.083) than for religiosity (*r* +  = 0.056). Although the difference is very small (Δ*r* +  = 0.031) and not significant, it may suggest that in German-speaking countries, where religious landscapes are characterized by ongoing secularization and possibly individualization (see, e.g., for Germany: Evangelische Kirche in Deutschland, 2023), measurement instruments are now being used that are better suited to assessing the meaning-making of the study populations. This is all the more true as the participants in the primary studies of the current meta-analysis are considerably younger (*M* = 31.4 years) than those in the previous meta-analysis (*M* = 43.5 years; Hodapp & Zwingmann, 2019, p. 1983). The more adequate inclusion of spirituality could therefore be one reason why the overall effect size is slightly higher in the updated meta-analysis than in the previous one (in which—albeit with a more general classification of the instruments—a lower average association with mental health was found for “spirituality” than for “centrality/salience/intrinsic” and “positive attitude towards religiosity/God”; Hodapp & Zwingmann, 2019, Fig. 2).

The content classification shows that the highest effect sizes are found for those aspects that are preferably operationalized with S scales (“experience,” “ideology,” and mixed forms including these aspects; 0.098 ≤ *r* +  ≤ 0.127). Smaller effect sizes are found for content aspects that are preferably operationalized with R scales (“salience/centrality,” “positive r/s coping/image/relationship,” and “practice”; 0.050 ≤ *r* +  ≤ 0.076). It is particularly striking that the aspect “positive r/s coping/image/relationship,” which had the relatively highest effect size in the previous meta-analysis (*r* +  = 0.07; Hodapp & Zwingmann, 2019, Fig. 2), is now at the lower end (*r* +  = 0.050). This could be an indication that the importance of religious coping has now decreased in favor of spiritual experience. Taken together, it can be concluded that in German-speaking countries, the (overall rather low) correlations between R/S and mental health are relatively strongest for spirituality. Study participants may experience such a reference to transcendence as a source of personal growth more strongly than religion.

### Limitations

A number of limitations must be considered when generalizing the findings of the present meta-analysis. The calculations are based on cross-sectional correlative data, which precludes the ability to draw causal conclusions. For example, it is not possible to decide whether certain forms of R/S promote mental health, or whether particular expressions of mental health give rise to specific forms of R/S, or whether there are some other variables that influence both R/S and mental health. It should also be noted that the bivariate effect sizes of the underlying primary studies are unable to account for any curvilinear relationships between R/S and mental health. Finally, as these are questionnaire data, response bias, such as social desirability, cannot be ruled out.

## Conclusions

In summary, the new meta-analysis of studies from the German-speaking area is characterized by the effort to differentiate the effects of religiosity and spirituality on mental health. To this end, the measurement instruments included in the primary studies were classified into R, S, and mixed scales (and also in terms of content) on the basis of a semantic analysis of all items. The results show that the average effect size is slightly higher for S and mixed scales than for R scales. This finding suggests that in the more secularized German-speaking world, it is more the non-institutionalized, conceptually and semantically open forms of transcendental meaning-making that are associated with mental health. These forms are presumably most strongly experienced as a source of personal growth.

The present meta-analysis shows a slightly higher overall effect size than the previous one. This is probably because of the fact that spirituality is now adequately included in many studies. Nevertheless, due to the magnitude of the overall effect size now found (*r* +  = 0.083), the conclusion of the previous meta-analysis must be confirmed: In German-speaking countries, the correlation between R/S and mental health tends to be low and is lower than in the United States.

In addition, the present meta-analysis for German-speaking countries revealed that the relationship between negative forms of R/S and mental health was not examined in general population samples in the period from 2016 to 2022/23, even though the previous meta-analysis had shown particularly high (negative) effect sizes in this area. This desideratum should be addressed in order to avoid an overly optimistic view of the importance of R/S for mental health in German-speaking countries.

## Supplementary Information

Below is the link to the electronic supplementary material.

Supplementary file1 (DOCX 13 kb)

Supplementary file2 (DOCX 53 kb)

## References

[CR1] *These are the primary studies which were included in the meta-analysis.

[CR2] Abdel-Khalek, A. M., Nuño, L., Gómez-Benito, J., & Lester, D. (2019). The relationship between religiosity and anxiety: A meta-analysis. *Journal of Religion and Health,**58*(5), 1847–1856. 10.1007/s10943-019-00881-z 31309442 10.1007/s10943-019-00881-z

[CR3] *Aderhold, C., Morawa, E., Paslakis, G., & Erim, Y. (2019). Entwicklung und Validierung eines Fragebogens zur Patientenkompetenz im Umgang mit einer Krebserkrankung (PUK). *Zeitschrift Für Psychosomatische Medizin und Psychotherapie,**65*(3), 239–256. 10.13109/zptm.2019.65.3.239 31476991 10.13109/zptm.2019.65.3.239

[CR4] *Alewell, D., Brinck, K. L., & Moll, T. (2022). (How) does religiousness impact on job satisfaction? Results for Germany. *Journal of Management, Spirituality & Religion,**19*(1), 21–44. 10.51327/TKJM6011

[CR5] Altman, D. G. (1991). Practical statistics for medical research. *Chapman & Hall*. 10.1201/9780429258589

[CR6] Baumann, K., Frick, E., Jacobs, C., & Büssing, A. (2019). Spiritual dryness and celibacy in Catholic priests: Discernment of ongoing spiritual journeys from relational and psychosexual immaturities. *Pastoral Psychology,**68*(6), 605–617. 10.1007/s11089-019-00886-1

[CR7] Bax, L., Ikeda, N., Fukui, N., Yaju, Y., Tsuruta, H., & Moons, K. G. M. (2008). More than numbers: The power of graphs in meta-analysis. *American Journal of Epidemiology,**169*(2), 249–255. 10.1093/aje/kwn340 19064649 10.1093/aje/kwn340

[CR8] Bertelsmann Stiftung (Ed.). (2008): *Religionsmonitor 2008. USA. Überblick zu religiösen Einstellungen und Praktiken*. Editor.

[CR9] Bertelsmann Stiftung (Ed.). (2022). *Religionsmonitor kompakt: Die Zukunft der Kirchen – zwischen Bedeutungsverlust und Neuverortung in einer vielfältigen Gesellschaft. Ergebnisse des Religionsmonitors 2023 – eine Vorschau*. Editor.

[CR10] Borenstein, M., Hedges, L. V., Higgins, J. P. T., & Rothstein, H. R. (2009). Introduction to meta-analysis. *Wiley*. 10.1002/9780470743386

[CR11] *Budimir, S., Probst, T., & Pieh, C. (2021). Coping strategies and mental health during COVID-19 lockdown. *Journal of Mental Health,**30*(2), 156–163. 10.1080/09638237.2021.1875412 33502917 10.1080/09638237.2021.1875412

[CR12] *Burri, A., Blank Gebre, M., & Bodenmann, G. (2017). Individual and dyadic coping in chronic pain patients. *Journal of Pain Research,**10*, 535–544. 10.2147/JPR.S128871 28331356 10.2147/JPR.S128871 PMC5349697

[CR13] Büssing, A., Baumann, K., Jacobs, C., & Frick, E. (2017). Spiritual dryness in Catholic priests: Internal resources as possible buffers. *Psychology of Religion and Spirituality,**9*(1), 46–55. 10.1037/rel0000063

[CR14] *Butz, S., Kieslich, P. J., & Bless, H. (2017). Why are conservatives happier than liberals? Comparing different explanations based on system justification, multiple group membership, and positive adjustment. *European Journal of Social Psychology,**47*(3), 362–372. 10.1002/ejsp.2283

[CR15] Cohen, J. (1988). *Statistical power analysis for the behavioral sciences* (2nd ed.). Erlbaum. 10.4324/9780203771587

[CR16] *Cwik, J. C., & Büssing, A. (2019). Spiritualität und Religiosität und ihr Zusammenhang mit Lebenszufriedenheit bei Personen mit Autismus-Spektrum-Störung. *Spiritual Care,**8*(3), 251–261. 10.1515/spircare-2018-0096

[CR17] *Edinger-Schons, L. M. (2020). Oneness beliefs and their effect on life satisfaction. *Psychology of Religion and Spirituality,**12*(4), 428–439. 10.1037/rel0000259

[CR18] Evangelische Kirche in Deutschland (Ed.). (2023). *Wie hältst du’s mit der Kirche? Zur Bedeutung der Kirche in der Gesellschaft. Erste Ergebnisse der 6. Kirchenmitgliedschaftsuntersuchung*. Evangelische Verlagsanstalt.

[CR19] Exline, J. J., Pargament, K. I., Wilt, J. A., Pait, K. C., & Schutt, W. A. (2024). Research on spiritual struggles: A brief snapshot focusing on new horizons. *Spiritual Care,**13*(2), 103–114. 10.1515/spircare-2022-0063

[CR20] Forouhari, S., Hosseini Teshnizi, S., Ehrampoush, M. H., Mazloomy Mahmoodabad, S. S., Fallahzadeh, H., Tabei, S. Z., et al. (2019). Relationship between religious orientation, anxiety, and depression among college students: A systematic review and meta-analysis. *Iranian Journal of Public Health,**48*(1), 43–52. https://pmc.ncbi.nlm.nih.gov/articles/PMC6401585/30847310 PMC6401585

[CR21] Garssen, B., Visser, A., & de Jager Meezenbroek, E. (2016). Examining whether spirituality predicts subjective well-being: How to avoid tautology. *Psychology of Religion and Spirituality,**8*(2), 141–148. 10.1037/rel0000025

[CR22] Garssen, B., Visser, A., & Pool, G. (2021). Does spirituality or religion positively affect mental health? Meta-analysis of longitudinal studies. *International Journal for the Psychology of Religion,**31*(1), 4–20. 10.1080/10508619.2020.1729570

[CR23] *Gebauer, J. E., Sedidikes, C., Schönbrodt, F. D., Bleidorn, W., Rentfrow, P. J., Potter, J., et al. (2017). The religiosity as social value hypothesis: A multi-method replication and extension across 65 countries and three levels of spatial aggregation. *Journal of Personality and Social Psychology,**113*(3), e18–e39. 10.1037/pspp0000104 27442765 10.1037/pspp0000104

[CR24] Hackney, C. H., & Sanders, G. S. (2003). Religiosity and mental health: A meta-analysis of recent studies. *Journal for the Scientific Study of Religion,**42*(1), 43–55. 10.1111/1468-5906.t01-1-00160

[CR25] *Hampel, N., Schauenburg, H., Ehrenthal, J. C., Brähler, E., Baie, L., & Heuft, G. (2019). Religiosität: In ihrem Einfluss auf ängstliche und depressive Symptome sowie Körperbeschwerden und Traumata überschätzt? Eine repräsentative Querschnittstudie. *Zeitschrift Für Psychosomatische Medizin und Psychotherapie,**65*(3), 288–303. 10.13109/zptm.2019.65.3.288 31476999 10.13109/zptm.2019.65.3.288

[CR26] *Hiebler-Ragger, M., Falthansl-Scheinecker, J., Birnhuber, G., Fink, A., & Unterrainer, H. F. (2016). Facets of spirituality diminish the positive relationship between insecure attachment and mood pathology in young adults. *PLoS ONE,**11*(6), e0158069. 10.1371/journal.pone.0158069 27336471 10.1371/journal.pone.0158069 PMC4919040

[CR27] Higgins, J. P. T., Thompson, S. G., Deeks, J. J., & Altman, D. G. (2003). Measuring inconsistency in meta-analyses. *British Medical Journal,**327*, 557–560. 10.1136/bmj.327.7414.557 12958120 10.1136/bmj.327.7414.557 PMC192859

[CR28] Hodapp, B., & Zwingmann, C. (2019). Religiosity/spirituality and mental health: A meta-analysis of studies from the German-speaking area. *Journal of Religion and Health,**58*(6), 1970–1998. 10.1007/s10943-019-00759-0 30632002 10.1007/s10943-019-00759-0

[CR29] *Höfer, S., Hausler, M., Huber, A., Strecker, C., Renn, D., & Höge, T. (2020). Psychometric characteristics of the German Values in Action Inventory 120-item short form. *Applied Research in Quality of Life,**15*, 597–611. 10.1007/s11482-018-9696-y 32457816 10.1007/s11482-018-9696-y PMC7250639

[CR30] *Huber, A., Strecker, C., Kachel, T., Höge, T., & Höfer, S. (2020). Character strength profiles in medical professionals and their impact on well-being. *Frontiers in Psychology,**11*, 566728. 10.3389/fpsyg.2020.566728 33424679 10.3389/fpsyg.2020.566728 PMC7786021

[CR31] Huber, S., & Huber, O. (2012). The centrality of religiosity scale (CRS). *Religions,**3*(3), 710–724. 10.3390/rel3030710

[CR32] Jeserich, F., Klein, C., Brinkhaus, B., & Teut, M. (2023). Sense of coherence and religion/spirituality: A systematic review and meta-analysis based on a methodological classification of instruments measuring religion/spirituality. *PLoS ONE,**18*(8), e0289203. 10.1371/journal.pone.0289203 37535597 10.1371/journal.pone.0289203 PMC10399782

[CR33] *Karwetzky, C., Michaelsen, M. M., Werdecker, L., & Esch, T. (2022). The U-curve of happiness revisited: Correlations and differences in life satisfaction over the span of life: An empirical evaluation based on data from 1,597 individuals aged 12–94 in Germany. *Frontiers in Psychology,**13*, 837638. 10.3389/fpsyg.2022.837638 35572301 10.3389/fpsyg.2022.837638 PMC9096900

[CR34] *Klein, C., & Bethe, S. (2021). Messbare Ausschnitte des Unermesslichen? Wie Spiritualität und spirituelles Wohlbefinden in der sozial- und gesundheitswissenschaftlichen Forschung gemessen werden können. In C. Richter (Ed.), *An den Grenzen des Messbaren. Die Kraft von Religiosität und Spiritualität in Lebenskrisen* (pp. 117–143). Kohlhammer.

[CR35] Klein, C., Gottschling, S., & Zwingmann, C. (2012). Deutschsprachige Fragebögen zur Messung von Religiosität/Spiritualität: Ein empirisch gestützter Vergleich ausgewählter Skalen. *Spiritual Care,**1*(3), 22–35. 10.1515/spircare-2012-0039

[CR36] *Klein, C., Keller, B., Silver, C. F., Hood, R. W., & Streib, H. (2016). Positive adult development and “spirituality”: Psychological well-being, generativity, and emotional stability. In H. Streib & R. W. Hood (Eds.), *Semantics and psychology of spirituality* (pp. 401–436). Springer. 10.1007/978-3-319-21245-6_25

[CR37] *Knorr, A., Podolin-Danner, N., Fuchshuber, J., Wenzl, M., Silani, G., & Unterrainer, H.-F. (2023). Development and validation of the Multidimensional Inventory for Religious/Spiritual Well-Being 18 item version (MI-RSWB-18). *Personality and Individual Differences,**209*, 112213. 10.1016/j.paid.2023.112213

[CR38] Koenig, H. G., & Carey, L. B. (2024). Religion, spirituality and health research: Warning of contaminated scales. *Journal of Religion and Health,**63*(5), 3729–3743. 10.1007/s10943-024-02112-6 39196443 10.1007/s10943-024-02112-6

[CR39] *Kralovec, K., Kunrath, S., Fartacek, C., Pichler, E.-M., & Plöderl, M. (2018). The gender-specific associations between religion/spirituality and suicide risk in a sample of Austrian psychiatric inpatients. *Suicide and Life-Threatening Behavior,**48*(3), 281–293. 10.1111/sltb.12349 28370188 10.1111/sltb.12349

[CR40] Lefevor, G. T., Davis, E. B., Paiz, J. Y., & Smack, A. C. P. (2021). The relationship between religiousness and health among sexual minorities: A meta-analysis. *Psychological Bulletin,**147*(7), 647–666. 10.1037/bul0000321 33793286 10.1037/bul0000321

[CR41] Pargament, K. I., & Exline, J. J. (2022). *Working with spiritual struggles in psychotherapy: From research to practice*. Guilford.

[CR42] *Peng-Keller, S., Moergeli, H., Hasenfratz, K., Naef, R., Rettke, H., Hefti, R., et al. (2021). Including the spiritual dimension in multimodal pain therapy: Development and validation of the Spiritual Distress and Resources Questionnaire (SDRQ). *Journal of Pain and Symptom Management,**62*(4), 747–756. 10.1016/j.jpainsymman.2021.02.021 33631326 10.1016/j.jpainsymman.2021.02.021

[CR43] Peterson, R. A., & Brown, S. P. (2005). On the use of beta coefficients in meta-analysis. *Journal of Applied Psychology,**90*(1), 175–181. 10.1037/0021-9010.90.1.175 15641898 10.1037/0021-9010.90.1.175

[CR44] Plöderl, M., Kunrath, S., & Fartacek, C. (2020). God bless you? The association of religion and spirituality with reduction of suicide ideation and length of hospital stay among psychiatric patients at risk for suicide. *Suicide and Life-Threatening Behavior,**50*(1), 95–110. 10.1111/sltb.12582 31410881 10.1111/sltb.12582

[CR45] *Proyer, R. T., & Laub, N. (2017). The german-language version of the Expressions of Spirituality Inventory-Revisited: Adaptation and initial validation. *Current Psychology: A Journal for Diverse Perspectives on Diverse Psychological Issues,**36*(1), 1–13. 10.1007/s12144-015-9379-x

[CR46] *Reissmann, M., Storms, A., & Woopen, C. (2021). Individual values and spirituality and their meaning for affective well-being and engagement with life in very old age. *Zeitschrift Für Gerontologie und Geriatrie,**54*(Suppl 2), S8–S92. 10.1007/s00391-021-01974-9 PMC8551090 34599384

[CR47] *Rojas, R., Hickmann, M., Wolf, S., Kolassa, I.-T., & Behnke, A. (2022). Coping in the emergency medical services: Associations with the personnel’s stress, self-efficacy, job satisfaction, and health. *Clinical Psychology in Europe,**4*(1), e6133. 10.32872/cpe.6133 36397746 10.32872/cpe.6133 PMC9667341

[CR48] Salsman, J. M., Pustejovsky, J. E., Jim, H. S. L., Munoz, A. R., Merluzzi, T. V., George, L., et al. (2015). A meta-analytic approach to examining the correlation between religion/spirituality and mental health in cancer. *Cancer,**121*(21), 3769–3778. https://doi.org/10.1002/cncr.29350 26258536 10.1002/cncr.29350 PMC4618157

[CR49] Sawatzky, R., Ratner, P. A., & Chiu, L. (2005). A meta-analysis of the relationship between spirituality and quality of life. *Social Indicators Research,**72*(2), 153–188. 10.1007/s11205-004-5577-x

[CR50] *Schlechter, P., Mateos Rodriguez, I., Morina, N., Knausenberger, J., Wilkinson, P. O., & Hellmann, J. H. (2021). Psychological distress in refugees: The role of traumatic events, resilience, social support, and support by religious faith. *Psychiatry Research,**304*, 114121. 10.1016/j.psychres.2021.114121 34303945 10.1016/j.psychres.2021.114121

[CR51] *Schmuck, J., Hiebel, N., Rabe, M., Schneider, J., Erim, Y., Morawa, E., et al. (2021). Sense of coherence, social support and religiosity as resources for medical personnel during the COVID-19 pandemic. A web-based survey among 4324 health care workers within the German Network University Medicine. *PLoS ONE,**16*(7), e0255211. 10.1371/journal.pone.0255211 34310616 10.1371/journal.pone.0255211 PMC8312980

[CR52] *Sorokowski, P., Randall, A. K., Groyecka, A., Frackowiak, T., Cantarero, K., Hilpert, P., et al. (2021). Marital Satisfaction, sex, age, marriage duration, religion, number of children, economic status, education, and collectivistic values: Data from 33 countries. *Frontiers in Psychology,**8*, 1199. 10.3389/fpsyg.2017.01199 10.3389/fpsyg.2017.01199 PMC5519603 28785230

[CR53] *Spitzenstätter, D., & Schnell, T. (2020). The existential dimension of the pandemic: Death attitudes, personal worldview, and coronavirus anxiety. *Death Studies,**46*(5), 1031–1041. 10.1080/07481187.2020.1848944 33357041 10.1080/07481187.2020.1848944

[CR54] *Stavrova, O., Ehlebracht, D., & Fetchenhauer, D. (2016). Belief in scientific-technological progress and life satisfaction: The role of personal control. *Personality and Individual Differences,**96*, 227–236. 10.1016/j.paid.2016.03.013

[CR55] *Surall, V., & Steppacher, I. (2020). How to deal with death: An empirical path analysis of a simplified model of death anxiety. *Omega-Journal of Death and Dying,**82*(2), 261–277. 10.1177/0030222818808145 10.1177/0030222818808145 30373472

[CR56] *Teismann, T., Willutzki, U., Michalak, J., Siegmann, P., Nyhuis, P., Wolter, M., et al. (2017). Religious beliefs buffer the impact of depression on suicide ideation. *Psychiatry Research,**257*, 276–278. 10.1016/j.psychres.2017.07.060 28783575 10.1016/j.psychres.2017.07.060

[CR57] *Vuzic, X. D., Burkart, P. L., Wenzl, M., Fuchshuber, J., & Unterrainer, H.-F. (2022). The relationship between religious/spiritual well-being, psychiatric symptoms and addictive behaviors among young adults during the COVID-19-pandemic. *Frontiers in Psychology,**13*, 942149. 10.3389/fpsyg.2022.942149 36172231 10.3389/fpsyg.2022.942149 PMC9511163

[CR58] Yaden, D. B., Batz-Barbarich, C. L., Ng, V., Vaziri, H., Gladstone, J. N., Pawelski, J. O., & Tay, L. (2022). A meta-analysis of religion/spirituality and life satisfaction. *Journal of Happiness Studies,**23*(8), 4147–4163. 10.1007/s10902-022-00558-7

[CR59] *Zacher, H., & Rudolph, C. W. (2021). Individual differences and changes in subjective wellbeing during the early stages of the COVID-19 pandemic. *American Psychologist,**76*(1), 50–62. 10.1037/amp0000702 32700938 10.1037/amp0000702

[CR60] Zwingmann, C. (2022). Religiosität und Lebensqualität. In M. Staats (Ed.), *Lebensqualität. Ein Metathema* (pp. 323–339). Beltz Juventa.

[CR61] Zwingmann, C., & Hodapp, B. (2018). Religiosität/Spiritualität und psychische Gesundheit: Zentrale Ergebnisse einer Metaanalyse über Studien aus dem deutschsprachigen Raum. *Spiritual Care,**7*(1), 69–80. 10.1515/spircare-2017-0019

[CR62] Zwingmann, C., Jeserich, F., & Büssing, A. (in press). Deutschsprachige Fragebögen zu Spiritualität/Religiosität: Semantische Analysen als Entscheidungshilfe zur Auswahl von Messinstrumenten. In E. Frick, T. Roser, & G. Stotz-Ingenlath (Eds.), *Spiritualität und Medizin. Gemeinsame Sorge für den kranken Menschen* (3rd rev. ed.). Kohlhammer.

[CR63] Zwingmann, C., & Klein, C. (2012). Deutschsprachige Fragebögen zur Messung von Religiosität/Spiritualität: Stellenwert. *Klassifikation und Auswahlkriterien. Spiritual Care,**1*(3), 7–21. 10.1515/spircare-2012-0038

[CR64] Zwingmann, C., Klein, C., & Büssing, A. (2011). Measuring religiosity/spirituality: Theoretical differentiations and categorization of instruments. *Religions,**2*(3), 345–357. 10.3390/rel2030345

[CR65] Zwingmann, C., Klein, C., & Jeserich, F. (2017). Religiosität: Die dunkle Seite“. Eine kurze Einführung. In C. Zwingmann, C. Klein, & F. Jeserich (Eds.), *Religiosität: Die dunkle Seite. Beiträge zur empirischen Religionsforschung* (pp. 11–19). Waxmann.

