# Supplementary material for: Religiosity, Spirituality and Mental Health: Meta-analysis of Studies from the German-Speaking Area
Source: J Relig Health. 2025 Aug 12;65(1):132–57. doi: 10.1007/s10943-025-02406-3 (PMC12913346; doi:10.1007/s10943-025-02406-3)
Supplement: Supplementary file 1 — Supplementary file1 (DOCX 13 kb) [file 10943_2025_2406_MOESM1_ESM.docx]

**Appendix 1** Boolean Search Strategy

**PSYNDEX, Di, 07 Mär 2023 18:50:58 MESZ**

(CM=10100 or CM=1011* or CM=10120)
[“empirische Studie allgemein” or “experimentelle Studie” or “Längsschnittstudie”]
AND
(Gott* OR beten OR Gebet OR Glaube OR gläubig OR relig* OR spirit*)
AND
(abhängig* OR Affekt OR Alkohol OR Angst* OR Belastung OR Depress* OR Drogen OR Erkrankung OR Gesundheit OR Glück OR Hoffnung* OR Krankheit OR Lebenszufriedenheit OR mental OR Optimis* OR Psych* OR Rauchen OR Resilienz OR Schuld* OR Selbstwert OR Stimmung OR Störung OR Stress* OR Substanzmissbrauch OR Suizid* OR Wachstum OR Wohlbefinden OR Zufriedenheit OR Zwang*))
AND
PY>=2015 PY<=2023 (**882 Treffer**)

**MEDLINE via PubMed, Tue May 02 11:56:34 2023**

(((god[Title/Abstract] OR pray*[Title/Abstract] OR faith[Title/Abstract] OR belief[Title/Abstract] OR church[Title/Abstract] OR relig*[Title/Abstract] OR spirit*[Title/Abstract])
AND
(addict*[Title] OR affect*[Title] OR alcohol[Title] OR anxiety[Title] OR burden[Title] OR depress*[Title] OR drug*[Title] OR health[Title] OR happiness[Title] OR hope*[Title] OR illness[Title] OR mental[Title] OR optimis*[Title] OR psychological[Title] OR smoking[Title] OR resilience[Title] OR guilt[Title] OR self-esteem[Title] OR mood[Title] OR disorder[Title] OR stress*[Title] OR substance use[Title] OR suicid*[Title] OR growth[Title] OR well-being[Title] OR satisfaction[Title] OR compuls*[Title] OR distress[Title]))
AND
(german*[Title/Abstract] OR austria*[Title/Abstract] OR Switzerland[Title/Abstract] OR swiss[Title/Abstract]))
AND
(("2015/01/01"[Date - Publication] : "3000"[Date - Publication])) (**239 items**)

**APA PsycNet (APA PsycInfo, APA PsycArticles, APA PsycBooks, APA PsycExtra),
Mo 08.05.2023 13:10**

Abstract: god OR Abstract: pray* OR Abstract: faith OR Abstract: belief OR Abstract: church OR Abstract: relig* OR Abstract: spirit*
AND
Abstract: addict* OR Abstract: affect* OR Abstract: alcohol OR Abstract: anxiety OR Abstract: burden OR Abstract: depress* OR Abstract: drug* OR Abstract: health OR Abstract: happiness OR Abstract: hope* OR Abstract: illness OR Abstract: mental OR Abstract: optimis* OR Abstract: psychological OR Abstract: smoking OR Abstract: resilience OR Abstract: guilt OR Abstract: self-esteem OR Abstract: mood OR Abstract: disorder OR Abstract: stress* OR Abstract: substance use OR Abstract: suicid* OR Abstract: growth OR Abstract: well-being OR Abstract: satisfaction OR Abstract: compuls* OR Abstract: distress

AND

Abstract: german* OR Abstract: austria* OR Abstract: Switzerland OR Abstract: swiss
AND
Methodology: Empirical Study

AND
Year: 2015 To 2023 (**261 items**)
