# Supplementary material for: Religiosity, Spirituality and Mental Health: Meta-analysis of Studies from the German-Speaking Area
Source: J Relig Health. 2025 Aug 12;65(1):132–57. doi: 10.1007/s10943-025-02406-3 (PMC12913346; doi:10.1007/s10943-025-02406-3)
Supplement: Supplementary file 2 — Supplementary file2 (DOCX 53 kb) [file 10943_2025_2406_MOESM2_ESM.docx]

**Appendix 2** Classification of R/S measurement instruments according to R/S and content

| (Sub)scale | Acronym | Key publication | Scale description | Study assessed for meta-analysis | Number of items | Item classification | R/S scale classification | Content scale classification |
| --- | --- | --- | --- | --- | --- | --- | --- | --- |
| Beten und Hoffen | CSQ-BuH | Original: Rosenstiel & Keefe (1983); German: Verra et al. (2006) | Subscale of the “Coping Strategies Questionnaire” (CSQ) | Burri et al. (2017) | 6 | X, R, X, X, RS, R | **X**  T: 50%  R: 50%  S: 17% | Not carried out |
| Cognitive Orientation Towards Spirituality | ESI-R – COS | Original: MacDonald (2000); German: Proyer & Laub (2017) | Subscale of the “Expressions of Spirituality Inventory-Revised” (ESI-R) | Proyer & Laub (2017) | 6 | S_1, S_1, S_1/2, S_1/2, S_1, S_1 | **S**  T: 100%  R: 0%  S: 100% | **1**: 100% Salience/ centrality |
| Connectedness (1) | MI-RSWB 48 – CO | Unterrainer et al. (2010) | Subscale of the “Multidimensional Inventory for Religious/Spiritual Well-Being” (MI-RSWB 48) | Knorr et al. (2023) | 8 | S_13, RS_12, S_13, S_13, SR_12, (S)_13, S_13, (S)_13 | **S**  T: 100%  R: 25%  S: 100% | **13**: 100% Experience |
| Connectedness (2) | MI-RSWB 18 – CO | Knorr et al. (2023) | Subscale of the “Multidimensional Inventory for Religious/Spiritual Well-Being 18 item version” (MI-RSWB 18) | Knorr et al. (2023) | 3 | RS_12, S_13, SR_12 | **SR**  T: 100%  R: 67%  S: 100% | **12**: 67% Ideology |
| Connectedness (3) | MI-RSWB 12 – CO | Fuchshuber & Unterrainer (2021) | Subscale of the “Multidimensional Inventory for Religious/Spiritual Well-Being Short Version” (MI-RSWB 12) | Vuzic et al. (2022) | 3 | S_13, S_13, S_13 | **S**  T: 100%  R: 0%  S: 100% | **13**: 100% Experience |
| Ehrfurcht/  Dankbarkeit (1) | SpREUK-P SF17 – ED | Büssing et al. (2012) | Subscale of the “Fragebogen zu Spirituellen und Religiösen Einstellungen im Umgang mit Krankheiten – Praktiken (17-Item-Version)” (SpREUK-P SF17) | Cwik & Büssing (2019) | 3 | (X), (SR), X | **X**  T: 33%  R: 33%  S: 33% | Not carried out |
| Ehrfurcht/ Dankbarkeit (2) | GrAw-7 | Büssing et al. (2018) | Scale without subscales | Büssing (2021, 2022), Büssing et al. (2021a, 2021b, 2022) | 7 | (X), (SR), X, (S), (S), RS, (X) | **X**  T: 57%  R: 29%  S: 57% | Not carried out |
| Existential Well-Being (1) | SWBS-EWB | Original: Paloutzian & Ellison (1982); German: Klein & Bethe (2021) | Subscale of the “Spiritual Well-Being Scale” (SWBS) | Klein & Bethe (2021) | 10 | (X), X, X, (X), X, X, X, X, (X), (S) | **X**  T: 10%  R: 0%  S: 10% | Not carried out |
| Existential Well-Being (2) | ESI-R – EWB | Original: MacDonald (2000); German: Proyer & Laub (2017) | Subscale of the “Expressions of Spirituality Inventory-Revised” (ESI-R) | Proyer & Laub (2017) | 6 | X, X, X, X, X, X | **X**  T: 0%  R: 0%  S: 0% | Not carried out |
| Existential Well-Being Score | MI-RSWB 48 – EWB | Unterrainer et al. (2010) | Dimension of the “Multidimensio­nal Inventory for Religious/Spiritual Well-being” (MI-RSWB 48) with three subscales: 1. Hope Immanent (8 items), 2. Forgiveness (8 items), 3. Experience of Sense and Meaning (8 items) | Hiebler-Ragger et al. (2016) | 24 | (X), (X), (X), (X), (X), (X), (X), (X), X, X, X, X, X, X, X, X, X, X, X, X, X, X, X, (S) | **X**  T: 4%  R: 0%  S: 4% | Not carried out |
| Existenzialistische Praktiken | SpREUK-P SF17 – EXP | Büssing et al. (2012) | Subscale of the “Fragebogen zu Spirituellen und Religiösen Einstellungen im Umgang mit Krankheiten – Praktiken (17-Item-version)” (SpREUK-P SF17) | Cwik & Büssing (2019) | 3 | S_14, S_14/13, S_14 | **S**  T: 100%  R: 0%  S: 100% | **14**: 100% Practice |
| Experiences of Sense and Meaning (1) | MI-RSWB 48 – SM | Unterrainer et al. (2010) | Subscale of the “Multidimensional Inventory for Religious/Spiritual Well-Being” (MI-RSWB 48) | Knorr et al. (2023) | 8 | X, X, X, X, X, X, X, (S) | **X**  T: 13%  R: 0%  S: 13% | Not carried out |
| Experiences of Sense and Meaning (2) | MI-RSWB 18 – SM | Knorr et al. (2023) | Subscale of the “Multidimensional Inventory for Religious/Spiritual Well-Being 18 item version” (MI-RSWB 18) | Knorr et al. (2023) | 3 | X, X, X | **X**  T: 0%  R: 0%  S: 0% | Not carried out |
| Experiential/Pheno-menological Dimen-sions of Spirituality | ESI-R – EPD | Original: MacDonald (2000); German: Proyer & Laub (2017) | Subscale of the “Expressions of Spirituality Inventory-Revised” (ESI-R) | Proyer & Laub (2017) | 6 | S_13, S_13, SR_13, S_13, SR/RS_13, S_13 | **S**  T: 100%  R: 33%  S: 100% | **13**: 100%  Experience |
| Extrovertierter Mystizismus | M-Skala – Extro | Original: Hood (1975); German: Streib et al. (2021) | Subscale of the “Mystizismus-Skala” (M-Skala) | Klein & Bethe (2021) | 8 | S_13*, S_13*, S_13, S_13, S_13*, S_13, S_13*, S_13 | **S**  T: 100%  R: 0%  S: 100% | **13**: 100%  Experience |
| Faith at Work Scale, German version | G-FAWS | Original: Lynn et al. (2013); German: Moll (2020) | Scale without subscales | Alewell et al. (2022) | 15 | RS/SR_13, RS/SR_5, (X), RS_1/2, RS_3, RS/SR_2, RS/SR_2, RS_2, RS/SR_2, RS/SR_2, RS_2, (X), (X), R_5, (R)_2 | **RS**  T: 80%  R: 80%  S: 67% | **2**: 53%  Consequences |
| Forgiveness (1) | MI-RSWB 48 – FO | Unterrainer et al. (2010) | Subscale of the “Multidimensional Inventory for Religious/Spiritual Well-Being” (MI-RSWB 48) | Knorr et al. (2023) | 8 | X, X, X, X, X, X, X, X | **X**  T: 0%  R: 0%  S: 0% | Not carried out |
| Forgiveness (2) | MI-RSWB 18 – FO | Knorr et al. (2023) | Subscale of the “Multidimensional Inventory for Religious/Spiritual Well-Being 18 item version” (MI-RSWB 18) | Knorr et al. (2023) | 3 | X, X, X | **X**  T: 0%  R: 0%  S: 0% | Not carried out |
| Forgiveness (3) | MI-RSWB 12 – FO | Fuchshuber & Unterrainer (2021) | Subscale of the “Multidimensional Inventory for Religious/Spiritual Well-Being Short Version” (MI-RSWB 12) | Vuzic et al. (2022) | 3 | X, X, X | **X**  T: 0%  R: 0%  S: 0% | Not carried out |
| Functional Assessment of Chronic Illness Therapy – Spiritual Well-Being | FACIT-Sp | Peterman et al. (2002) | Scale with two subscales: 1. Meaning/Peace (12 items), 2. Faith (4 items) | Grünke et al. (2018), Philipp et al. (2017), Scheffold et al. (2019) | 12 | (X), (X), X, (X), S, (S), (S), S, SR, SR, SR, (X) | **X**  T: 58%  R: 25%  S: 58% | Not carried out |
| General Religiosity (1) | MI-RSWB 48 – GR | Unterrainer et al. (2010) | Subscale of the “Multidimensional Inventory for Religious/Spiritual Well-Being” (MI-RSWB 48) | Knorr et al. (2023) | 8 | RS_3, R_14/3, R_3, R_13/3, R_3, R_3, R_14, RS_13 | **R**  T: 100%  R: 100%  S: 25% | **3**: 75%  Positive r/s coping/image/relationship |
| General Religiosity (2) | MI-RSWB 18 – GR | Knorr et al. (2023) | Subscale of the “Multidimensional Inventory for Religious/Spiritual Well-Being 18 item version” (MI-RSWB 18) | Knorr et al. (2023) | 3 | R_3, R_13/3, R_3 | **R**  T: 100%  R: 100%  S: 0% | **3**: 100%  Positive r/s coping/image/relationship |
| General Religiosity (3) | MI-RSWB 12 – GR | Fuchshuber & Unterrainer (2021) | Subscale of the “Multidimensional Inventory for Religious/Spiritual Well-Being Short Version” (MI-RSWB 12) | Vuzic et al. (2022) | 3 | RS_3, R_14/3, R_3 | **R**  T: 100%  R: 100%  S: 33% | **3**: 100%  Positive r/s coping/image/relationship |
| Greater Good Motivations | WAMI – GG | Original: Steger et al. (2012); German: Harzer (2016) | Subscale of the “Work and Meaning Inventory” (WAMI) | Listopad et al. (2021) | 3 | X, X, S | **X**  T: 33%  R: 0%  S: 33% | Not carried out |
| Halt im Glauben | SCI-HiG | Satow (2012) | Subscale of the “Stress- und Copinginventar” (SCI) | Budimir et al. (2021); Schlechter et al. (2021) | 4 | RS_3, RS_3/14, S_3/12, (X) | **SR**  T: 75%  R: 50%  S: 75% | **3**: 100%  Positive r/s coping/image/relationship |
| Häufigkeit Kirchgang | - | ALLBUS 2010 | Single item | Butz et al. (2017) | 1 | R_14 | **R**  T: 100%  R: 100%  S: 0% | **14**: 100%  Practice |
| Hope | MI-RSWB 12 – HO | Fuchshuber & Unterrainer (2021) | Subscale of the “Multidimensional Inventory for Religious/Spiritual Well-Being Short Version” (MI-RSWB 12) | Vuzic et al. (2022) | 3 | (X), (X), (X) | **X**  T: 0%  R: 0%  S: 0% | Not carried out |
| Hope Immanent (1) | MI-RSWB 48 – HI | Unterrainer et al. (2010) | Subscale of the “Multidimensional Inventory for Religious/Spiritual Well-Being” (MI-RSWB 48) | Knorr et al. (2023) | 8 | (X), (X), (X), (X), (X), (X), (X), (X) | **X**  T: 0%  R: 0%  S: 0% | Not carried out |
| Hope Immanent (2) | MI-RSWB 18 – HI | Knorr et al. (2023) | Subscale of the “Multidimensional Inventory for Religious/Spiritual Well-Being 18 item version” (MI-RSWB 18) | Knorr et al. (2023) | 3 | (X), (X), (X) | **X**  T: 0%  R: 0%  S: 0% | Not carried out |
| Hope Transcendent (1) | MI-RSWB 48 – HT | Unterrainer et al. (2010) | Subscale of the “Multidimensional Inventory for Religious/Spiritual Well-Being” (MI-RSWB 48) | Knorr et al. (2023) | 8 | X, X, X, X, X, (RS), X, (RS) | **X**  T: 25%  R: 25%  S: 25% | Not carried out |
| Hope Transcendent (2) | MI-RSWB 18 – HT | Knorr et al. (2023) | Subscale of the “Multidimensional Inventory for Religious/Spiritual Well-Being 18 item version” (MI-RSWB 18) | Knorr et al. (2023) | 3 | X, X, X | **X**  T: 0%  R: 0%  S: 0% | Not carried out |
| Interpretation | M-Skala – Inter | Original: Hood (1975); German: Streib et al. (2021) | Subscale of the “Mystizismus-Skala” (M-Skala) | Klein & Bethe (2021) | 12 | (X), (S)_13*, SR/RS_13*, (S)_13/12, RS/SR_13*, S_13*, SR_13, S_13, SR/RS_13/12, (S)_13, (S)_13*, (S)_13* | **S**  T: 92%  R: 33%  S: 92% | **13**: 92%  Experience |
| Introvertierter Mystizismus | M-Skala – Intro | Original: Hood (1975); German: Streib et al. (2021) | Subscale of the “Mystizismus-Skala” (M-Skala) | Klein & Bethe (2021) | 12 | S_13, (S)_13*, S_13, (S)_13, S_13-, S_13, S_13*, (S)_13*, (S)_13, SR_13*, S_13*, (S)_13 | **S**  T: 100%  R: 8%  S: 100% | **13**: 100%  Experience |
| Meaning Making through Work | WAMI – MM | Original: Steger et al. (2012); German: Harzer (2016) | Subscale of the “Work and Meaning Inventory” (WAMI) | Listopad et al. (2021) | 3 | (S), (X), (X) | **X**  T: 33%  R: 0%  S: 33% | Not carried out |
| Multidimensional Inventory for Religious/Spiritual Well-being | MI-RSWB 48 | Unterrainer et al. (2010) | Scale with six subscales: 1. General Religiosity (8 items), 2. For­giveness (8 items), 3. Hope Immanent (8 items), 4. Connectedness (8 items), 5. Hope Transcendent (8 items), 6. Experiences of Sense and Meaning (8 items) | Hiebler-Ragger et al. (2016) | 48 | RS, X, (X), S, X, X, R, X, (X), RS, X, X, R, X, (X), S, X, X, R, X, (X), S, X, X, R, X, (X), SR, X, X, R, X, (X), (S), (RS), X, R, X, (X), S, (X), X, RS, X, (X), (S), (RS), (SS_13-, ) | **X**  T: 40%  R: 25%  S: 27% | Not carried out |
| Multidimensional Inventory for Religious/Spiritual Well-being 18 item version | MI-RSWB 18 | Knorr et al. (2023) | Scale with six subscales: 1. General Religiosity (3 items), 2. For­giveness (3 items), 3. Hope Immanent (3 items), 4. Connectedness (3 items), 5. Hope Transcendent (3 items), 6. Experiences of Sense and Meaning (3 items) | Knorr et al. (2023) | 18 | R, X, (X), RS, X, X, R, X, (X), S, X, X, R, X, (X), SR, X, X | **X**  T: 33%  R: 28%  S: 17% | Not carried out |
| Mystizismus-Skala | M-Skala | Original: Hood (1975); German: Streib et al. (2021) | Scale with three subscales: 1. Introvertierter Mystizismus (12 items), 2. Extrovertierter Mystizismus (8 items),  3. Interpretationen (12 items) | Klein & Bethe (2021) | 32 | S_13, (S)_13*, S_13, (S)_13, (X), S_13*, (S)_13*, S_13*, SR/RS_13*, S_13*, S_13, S_13, (S)_13/12, RS/SR_13*, S_13*, S_13*, SR_13, S_13, S_13, SR/RS_13/12, (S)_13*, (S)_13, (S)_13, SR_13*, (S)_13*, (S)_13*, S_13*, S_13*, S_13, S_13*, S_13, (S)_13 | **S**  T: 97%  R: 16%  S: 97% | **13**: 97%  Experience |
| Öffentliche Religiöse Praxis (1) | FRA-RE, item 3 | Heuft (2016) | Single item taken from the “Fragebogen zu religiösen Einstellungen” (FRA-RE) | Hampel et al. (2019) | 1 | RS_14 | **RS**  T: 100%  R: 50%  S: 50% | **14**: 100%  Practice |
| Öffentliche Religiöse Praxis (2) | Z-10 – ÖPraxis | Huber (2003, 2004), Huber & Huber (2012) | Subscale of the “Zentralitätsskala (10-Item-Version)” (Z-10) | Kralovec et al. (2018) | 2 | R_14, R_14 | **R**  T: 100%  R: 100%  S: 0% | **14**: 100%  Practice |
| Oneness Beliefs Scale | OBS | Edinger-Schons (2020) | Scale without subscales | Edinger-Schons (2020) | 5 | S_12, SR_12, S_12, RS_12, (S)_12 | **S**  T: 100%  R: 29%  S: 100% | **12**: 100%  Ideology |
| Paranormal Beliefs | ESI-R – PAR | Original: MacDonald (2000); German: Proyer & Laub (2017) | Subscale of the “Expressions of Spirituality Inventory-Revised” (ESI-R) | Proyer & Laub (2017) | 6 | S_12, SR_12, (S)_12, SR_12, S_12, S_12 | **S**  T: 100%  R: 33%  S: 100% | **12**: 100% Ideology |
| Positive Meaning | WAMI – PM | Original: Steger et al. (2012); German: Harzer (2016) | Subscale of the “Work and Meaning Inventory” (WAMI) | Listopad et al. (2021) | 4 | (X), (S), (X), X | **X**  T: 25%  R: 0%  S: 25% | Not carried out |
| Private Religiöse Praxis | Z-10 – PPraxis | Huber (2003, 2004), Huber & Huber (2012) | Subscale of the “Zentralitätsskala (10-Item-Version)” (Z-10) | Kralovec et al. (2018) | 2 | RS_14, RS_14 | **RS/SR**  T: 100%  R: 100%  S: 100% | **14**: 100%  Practice |
| Prosozial-humanistische Praktiken | SpREUK-P SF17 – PSHP | Büssing et al. (2012) | Subscale of the “Fragebogen zu Spirituellen und Religiösen Einstellungen im Umgang mit Krankheiten – Praktiken (17-Item-Version)” (SpREUK-P SF 17) | Cwik & Büssing (2019) | 4 | X, X, X, X | **X**  T: 0%  R: 0%  S: 0% | Not carried out |
| Questionnaire on Spirituality, Item 1_1 | QueSt, Item 1_1 | Study NRW80+ | Single item taken from the “Questionnaire on Spirituality” (QueSt) | Reissmann et al. (2021) | 1 | RS_13/3 | **RS**  T: 100%  R: 100%  S: 100% | **13**: 100%  Experience  **3**: 100% Positive r/s coping/image/relationship |
| Questionnaire on Spirituality, Item 2_1 | QueSt, Item 2_1 | Study NRW80+ | Single item taken from the “Questionnaire on Spirituality” (QueSt) | Reissmann et al. (2021) | 1 | S_13 | **S**  T: 100%  R: 0%  S: 100% | **13**: 100%  Experience |
| Questionnaire on Spirituality, Item 3_1 | QueSt, Item 3_1 | Study NRW80+ | Single item taken from the “Questionnaire on Spirituality” (QueSt) | Reissmann et al. (2021) | 1 | S_13 | **S**  T: 100%  R: 0%  S: 100% | **13**: 100%  Experience |
| Questionnaire on Spirituality, Item 4_1 | QueSt, Item 4_1 | Study NRW80+ | Single item taken from the “Questionnaire on Spirituality” (QueSt) | Reissmann et al. (2021) | 1 | RS_14 | **RS**  T: 100%  R: 100%  S: 100% | **14**: 100%  Practice |
| Questionnaire on Spirituality, Item 5_1 | QueSt, Item 5_1 | Study NRW80+ | Single item taken from the “Questionnaire on Spirituality” (QueSt) | Reissmann et al. (2021) | 1 | SR_12 | **SR**  T: 100%  R: 100%  S: 100% | **12**: 100%  Ideology |
| Questionnaire on Spirituality, Item 6_1 | QueSt, Item 6_1 | Study NRW80+ | Single item taken from the “Questionnaire on Spirituality” (QueSt) | Reissmann et al. (2021) | 1 | RS_14 | **RS**  T: 100%  R: 100%  S: 100% | **14**: 100%  Practice |
| Questionnaire on Spirituality, Item 8 | QueSt, Item 7_1 | Study NRW80+ | Single item taken from the “Questionnaire on Spirituality” (QueSt) | Reissmann et al. (2021) | 1 | RS_1 | **RS**  T: 100%  R: 100%  S: 100% | **1**: 100%  Salience/centrality |
| Religiöse Erfahrung | Z-10 – Erfahrung | Huber (2003, 2004), Huber & Huber (2012) | Subscale of the “Zentralitätsskala (10-Item-Version)” (Z-10) | Kralovec et al. (2018) | 2 | RS_13, RS_13 | **RS/SR**  T: 100%  R: 100%  S: 100% | **13**: 100%  Experience |
| Religiöse Ideologie | Z-10 – Ideologie | Huber (2003, 2004), Huber & Huber (2012) | Subscale of the “Zentralitätsskala (10-Item-Version)” (Z-10) | Kralovec et al. (2018) | 2 | RS_12, SR_12 | **RS/SR**  T: 100%  R: 100%  S: 100% | **12**: 100%  Ideology |
| Religiöse Praktiken | SpREUK-P SF17 – RP | Büssing et al. (2012) | Subscale of the “Fragebogen zu Spirituellen und Religiösen Einstellungen im Umgang mit Krankheiten – Praktiken (17-Item-Version)” (SpREUK-P SF17) | Cwik & Büssing (2019) | 4 | RS_14, R_14, R_14, R_14 | **R**  T: 100%  R: 100%  S: 25% | **14**: 100%  Practice |
| Religiöse Selbsteinschätzung (1) | - | Moll (2020) | Single item | Alewell et al. (2022) | 1 | R_1 | **R**  T: 100%  R: 100%  S: 0% | **1**: 100%  Salience/centrality |
| Religiöse Selbsteinschätzung (2) | - | ALLBUS 2010 | Single item | Butz et al. (2017) | 1 | R_1 | **R**  T: 100%  R: 100%  S: 0% | **1**: 100%  Salience/centrality |
| Religiöse Selbsteinschätzung (3) | SIRS | Original:  Norenzayan & Hansen (2006) | Single item, variant of the “Single Item Religiosity Scale” (SIRS) | Gebauer et al. (2017) | 1 | R_1 | **R**  T: 100%  R: 100%  S: 0% | **1**: 100%  Salience/centrality |
| Religiöse  Selbsteinschätzung (4) | FRA-RE, Item 1 | Heuft (2016) | Single item taken from the “Fragebogen zu religiösen Einstellungen” (FRA-RE) | Hampel et al. (2019) | 1 | R_1/11* | **R**  T: 100%  R: 100%  S: 0% | **11**: 100%  Interest |
| Religiöse Selbsteinschätzung (5) | - | - | Single item | Karwetzky et al. (2022) | 1 | RS_1 | **RS**  T: 100%  R: 100%  S: 100% | **1**: 100% Salience/centrality |
| Religiöse Selbsteinschätzung (6) | TPV-11, item 1 | Belschner (1999) | Single item taken from the scale “Trans­personales Vertrauen” (TPV-11) | Schmuck et al. (2021) | 1 | SR/RS_3 | **SR/RS**  T: 100%  R: 100%  S: 100% | **3**: 100%  Positive r/s coping/image/relationship |
| Religiöse Selbsteinschätzung (7) | - | - | Single item | Sorokowski et al. (2017) | 1 | R_1 | **R**  T: 100%  R: 100%  S: 0% | **1**: 100%  Salience/centrality |
| Religiöse/Spirituelle Praxis | FRA-RE, Item 4 | Heuft (2016) | Single item taken from the “Fragebogen zu religiösen Einstellungen” (FRA-RE) | Hampel et al. (2019) | 1 | RS_14 | **RS**  T: 100%  R: 50%  S: 50% | **14**: 100%  Practice |
| Religiöses Interesse | Z-10 – Interesse | Huber (2003, 2004), Huber & Huber (2012) | Subscale of the “Zentralitätsskala (10-Item-Version)” (Z-10) | Kralovec et al. (2018) | 2 | RS_11, RS_11 | **RS/SR**  T: 100%  R: 100%  S: 100% | **11**: 100%  Interest |
| Religiöses/Spirituelles Coping | PUK-RSC | Aderhold et al. (2019) | Subscale of the “Fragebogen zur Patientenkompetenz im Umgang mit einer Krebserkrankung” (PUK) | Aderhold et al. (2019) | 4 | RS_3, RS_3, RS/SR_3, RS/SR_3 | **RS/SR**  T: 100%  R: 100%  S: 100% | **3**: 100%  Positive r/s coping/image/relationship |
| Religiosität (1) | LEBE – R | Schnell & Becker (2007) | Subscale of the “Fragebogen zu Lebensbedeutungen und Lebenssinn” (LEBE) | Spitzenstätter & Schnell (2020) | 3 | R_1, RS_14, RS_3/1 | **RS**  T: 100%  R: 100%  S: 67% | **1**: 100% Salience/centrality |
| Religiosität (2) | - | World Values Survey | Two single items, combined into one indicator | Stavrova et al. (2016) | 2 | R_14, R_1 | **R**  T: 100%  R: 100%  S:0% | **1**: 100%  Salience/centrality |
| Religiosität (3) | - | Surall & Steppacher (2020) | Scale without subscales | Surall & Steppacher (2020) | 7 | R_12, R_12, R_12, R_12, R_1, S_1, RS/SR_12* | **R**  T: 100%  R: 86%  S: 29% | **12**: 71%  Ideology |
| Religiosität und Sinnsuche | FKV-LIS – RuS | Muthny (1989) | Subscale of the “Freiburger Fragebogens zur Krankheitsverarbeitung” (FKV-LIS), short form | Hütter et al. (2020) | 5 | RS, R, S, X, X | **X**  T:60%  R: 50%  S: 50% | Not carried out |
| Religious Coping (1) | COPE-RC – Skala 9 | Original: Carver et al. (1989) | Subscale of the “Measure of Coping Styles and Strategies” (COPE) | Burri et al. (2017) | 4 | R_3, R_3, R_3, RS_3/14 | **R**  T: 100%  R: 100%  S: 25% | **3**: 100%  Positive r/s coping/image/relationship |
| Religious Coping (2) | Brief-COPE German – Skala 14 | Original: Carver (1997); German: Knoll et al. (2005) | Subscale of the “Brief Coping Orientation to Problems Experienced Inventory” (Brief-COPE) | Rojas et al. (2022); Zacher & Rudolph (2021) | 2 | RS_3, RS/SR_14/3 | **RS**  T: 100%  R: 100%  S: 100% | **3**: 100%  Positive r/s coping/image/relationship |
| Religiousness | ESI-R –  REL | Original: MacDonald (2000); German: Proyer & Laub (2017) | Subscale of the “Expressions of Spirituality Inventory-Revised” (ESI-R) | Proyer & Laub (2017) | 6 | R_14, SR_13/3, R_1, RS_1/2, RS_14, RS/SR_12 | **RS**  T: 100%  R: 100%  S: 67% | **1**: 100% Salience/centrality |
| Religious Well-Being | SWBS-RWB | Original: Paloutzian & Ellison (1982); German: Klein & Bethe (2021) | Subscale of the “Spiritual Well-Being Scale” (SWBS) | Klein & Bethe (2021) | 10 | R_3*, R_3, R_4, R_3, R_3*, R_3, R_3*, R_3, R_3, R_3 | **R** T: 100%  R: 100%  S: 0% | **5**: 80%  Positive r/s coping/image/relationship |
| Religious Well-Being Score | MI-RSWB 48 – RWB | Unterrainer et al. (2010) | Dimension of the “Multidimensional Inventory for Religious/Spiritual Well-being” (MI-RSWB 48) with three subscales: 1. Hope Transcendent (8 items), 2. General Religiosity (8 items), 3. Connectedness (8 items) | Hiebler-Ragger et al. (2016) | 24 | X, X, X, X, X, (RS)_3*, X, (RS)_3*, RS_3, R_14/3, R_3, R_13/3, R_3, R_3, R_14, RS_13, S_13, RS_12, S_13, S_13, SR_12, (S)_13, S_13, (S)_13 | **RS**  T: 75%  R: 50%  S: 50% | **1**: 67%  Salience/centrality |
| Self-Transcendence Scale | TCI-STS | Cloninger (1994) | Subscale of the “Temperament and Character Inventory” (TCI) | Kralovec et al. (2018) | 15 | (S)_13, S_13, S_13, (X), S_13, (S)_13, S_13, S_13, S_13/3, (X), (X), SR_13/3, S_13, S_12, (X) | **S**  T: 73%  R: 7%  S: 73% | **13**: 67%  Experience |
| Spiritualität (1) | VIA-120 – Sp | Original: Littmann-Ovadia (2015) | Subscale of the “Values in Action Inventory of Strengths” (VIA-120) | Höfer et al. (2020) | 5 | R_14, RS_1, RS_1, SR_12, R_1/12 | **RS**  T: 100%  R: 100%  S: 60% | **1**: 100% Salience/centrality |
| Spiritualität (2) | VIA-IS – Sp | Original: Peterson & Seligman (2004); German: Ruch et al. (2010) | Subscale of the “Values in Action Inventory of Strengths” (VIA-IS, 240 items) | Huber et al. (2020) | 10 | S_1, R_14, RS_1, (X), RS_14, RS_1, (X), SR_12, R_1/12, (X) | **RS**  T: 70%  R: 60%  S: 50% | **1**: 70% Salience/centrality |
| Spiritualität (3) | LEBE – Sp | Schnell & Becker (2007) | Subscale of the “Fragebogen zu Lebensbedeutungen und Lebenssinn” (LEBE) | Spitzenstätter & Schnell (2020) | 5 | RS_12, RS_12, RS_12, S_12, S_12/13 | **SR**  T: 100%  R: 60%  S: 100% | **12**: 100%  Ideology |
| Spiritual Coping, rearranged | SDRQ-SC, new | Peng-Keller et al. (2021) | Subscale of the “Spiritual Distress and Resources Questionnaire” (SDRQ) | Peng-Keller et al. (2021) | 3 | X, X, (X) | **X**  T: 0%  R: 0%  S: 0% | Not carried out |
| Spiritual Distress | SDRQ-SD | Peng-Keller et al. (2021) | Subscale of the “Spiritual Distress and Resources Questionnaire” (SDRQ) | Peng-Keller et al. (2021) | 8 | (S), (X), (X), X, X, RS, X, X | **X**  T: 25%  R: 13%  S: 25% | Not carried out |
| Spiritual Resources Immanence, rearranged | SDRQ-SRI, new | Peng-Keller et al. (2021) | Subscale of the “Spiritual Distress and Resources Questionnaire” (SDRQ) | Peng-Keller et al. (2021) | 4 | S, (X), (X), (X) | **X**  T: 25%  R: 0%  S: 25% | Not carried out |
| Spiritual Resources Transcendence, rearranged | SDRQ-SRT, new | Peng-Keller et al. (2021) | Subscale of the “Spiritual Distress and Resources Questionnaire” (SDRQ) | Peng-Keller et al. (2021) | 3 | S_13/3, S_13, RS_14 | **S**  T: 100%  R: 33.3%  S: 100% | **13**: 67%  Experience |
| Spiritual Well-Being Scale | SWBS | Original: Paloutzian & Ellison (1982); German: Klein & Bethe (2021) | Scale with two subscales: 1. Religious Well-Being (10 items), 2. Existential Well-Being (10 items) | Klein & Bethe (2021) | 20 | R, (X), R, X, R, X, R, (X), R, X, R, X, R, X, R, X, R, (X) R, (S) | **X**  T: 55%  R: 50%  S: 5% | Not carried out |
| Spirituelle Geist/Körper -Praktiken | SpREUK-P SF17 – SKGP | Büssing et al. (2012) | Subscale of the “Fragebogen zu Spirituellen und Religiösen Einstellungen im Umgang mit Krankheiten – Praktiken (17-Item-Version)” (SpREUK-P SF17) | Cwik & Büssing (2019) | 3 | SR_14, S_14, SR_14 | **SR**  T: 100%  R: 50%  S: 100% | **141**: 100% Practice |
| Spirituelle Selbsteinschätzung | FRA-RE, Item 2 | Heuft (2016) | Single item taken from the “Fragebogen zu religiösen Einstellungen” (FRA-RE) | Hampel et al. (2019) | 1 | S_1 | **S**  T: 100%  R: 0%  S: 100% | **1**: 100% Salience/centrality |
| Transpersonales Vertrauen revidiert | TPV-8R | Albani et al. (2003) | Scale with two subscales: 1. Transzendente Führung (4 items), 2. Transzendente Eingebundenheit (4 items) | Klein & Bethe (2021) | 8 | SR_3, S_3, SR_3, S_12/13, S_12/13, (S)_12, SR_3, S_13 | **S**  T: 100%  R: 38%  S: 100% | **5**: 50%  Positive r/s coping/image/relationship |
| Transzendente Eingebundenheit | TPV-8R-E | Albani et al. (2003) | Subscale of the scale “Transpersonales Vertrauen revidiert” (TPV-8R) | Klein & Bethe (2021) | 4 | S_12/13, S_12/13, (S)_12, S_13 | **S**  T: 100%  R: 0%  S: 100% | **12**: 75%  Ideology  **13**: 75%  Experience |
| Transzendente Führung | TPV-8R-F | Albani et al. (2003) | Subscale of the scale “Transpersonales Vertrauen revidiert” (TPV-8R) | Klein & Bethe (2021) | 4 | SR_3, SR_3, SR_3, S_3 | **SR**  T: 100%  R: 75%  S: 100% | **3**: 100% Positive r/s coping/image/relationship |
| Trust in Higher Guidance/Source | SpREUK-15 – THG | Büssing (2010) | Subscale of the scale “Spirituelle und Religiöse Einstellungen im Umgang mit Krankheit” (SpREUK-15) | Teismann et al. (2017) | 5 | S_3, R_1, SR_3, S_3, RS/SR_12 | **SR**  T: 100%  R: 60%  S: 80% | **5**: 60%  Positive r/s coping/image/relationship |
| Turning to Social and Religious Resources | COSS-SRR | Krohne et al. (2000) | Subscale of the “Coping with Surgical Stress Scale” (COSS) | Goebel et al. (2018) | 6 | RS, R, X,X, X, X | **X**  T: 33%  R: 33%  S: 17% | Not carried out |
| Work and Meaning Inventory | WAMI | Original: Steger et al. (2012); German: Harzer (2016) | Scale with three subscales: 1. Positive Meaning (4 items), 2. Meaning Making through Work (3 items), 3. Greater Good Motivations (3 items) | Listopad et al. (2021) | 10 | (X), (S), X, (S), (X), X, (X), X, (X), S | **X**  T: 30%  R: 0%  S: 30% | Not carried out |
| Zentralitätsskala (10-Item-Version) | Z-10 | Huber (2003, 2004), Huber & Huber (2012) | Scale with five subscales: 1. Interesse (2 items), 2. Ideologie (2 items), 3. Öffentliche Praxis (2 items), 4. Private Praxis (2 items), 5. Erfahrung (2 items) | Kralovec et al. (2018) | 10 | RS_11, RS_12, R_14, RS_14, RS_13, RS_11, SR_12, R_14, RS_14, RS_13 | **RS**  T: 100%  R: 100%  S: 80% | **1**: 100%  Salience/centrality |

R = primarily religious; (R) = not clearly religious, but can be interpreted religiously; RS = religious with strong spiritual components; RS/SR resp. SR/RS = religious and spiritual aspects in approximately equal proportions; S = primarily spiritual; (S) = not clearly spiritual, but can be interpreted spiritually; SR = spiritual with strong religious components; T = proportion of items in the scale measuring (religious or spiritual) transcendence (%); X = neither religious nor spiritual aspects; (X) = neither religious nor spiritual aspects, but the item could possibly be interpreted religiously/spiritually; * = the item measures the absence of the construct; 1 = salience/centrality; 11 = interest; 12 = ideology; 13 = experience; 14 = practice; 2 = consequences; 3 = positive religious/spiritual coping/image/relationship; 4 = negative religious/spiritual coping/image/relationship.

**Additional References**

Albani, C., Bailer, H., Blaser, G., Geyer, M., Brähler, E., & Grulke, N. (2003). Psychometrische Überprüfung der Skala “Transpersonales Vertrauen” (TPV) in einer repräsentativen Bevölkerungsstichprobe. *Transpersonale Psychologie und Psychotherapie, 9*, 86–98. <https://doi.org/10.1055/a-1033-7627>

Belschner, W. (1999). *Die Skala Transpersonales Vertrauen TPV* (Transpersonale Arbeitspapiere, Nr. 2). Universität Oldenburg.

Büssing, A. (2010). Spirituality as a resource to rely on in chronic illness. The SpREUK questionnaire. *Religions, 1*(1), 9–17. <https://doi.org/10.3390/rel1010009>

Büssing, A. (2021). Wondering awe as a perceptive aspect of spirituality and its relation to indicators of wellbeing: Frequency of perception and underlying triggers. *Frontiers in Psychiatry, 12*, Article 738770. <https://doi.org/10.3389/fpsyg.2021.738770>

Büssing, A. (2022). Veränderungen im Erleben von Ehrfurcht und Dankbarkeit während der Coronapandemie. *Bewusstseinswissenschaften, 28*(2), 6–14.

Büssing, A., Recchia, D. R., & Baumann, K. (2018). Validation of the Gratitude/Awe Questionnaire and its association with disposition of gratefulness. *Religions, 9*(4), 117. <https://doi.org/10.3390/rel9040117>

Büssing, A., Recchia, D. R., & Baumann, K. (2022). Experience of nature and times of silence as a resource to cope with the COVID-19 pandemic and their effects on psychological wellbeing – Findings from a continuous cross-sectional survey in Germany. *Frontiers in Public Health, 10*, Article 1020053. <https://doi.org/10.3389/fpubh.2022.1020053>

Büssing, A., Recchia, D. R., Dienberg, T., Surzykiewicz, J., & Baumann, K. (2021a). Awe/gratitude as an experiential aspect of spirituality and its association to perceived positive changes during the COVID-19 pandemic. *Frontiers in Psychiatry, 12*, Article 642716. <https://doi.org/10.3389/fpsyt.2021.642716>

Büssing, A., Recchia, D. R., Dienberg, T., Surzykiewicz, J., & Baumann, K. (2021b). Dynamics and perceived positive changes and indicators of well-being within different phases of the COVID-19 pandemic. *Frontiers in Psychiatry, 12*, Article 685975. <https://doi.org/10.3389/fpsyt.2021.685975>

Büssing, A., Reiser, F., Michalsen, A., & Baumann, K. (2012). Engagement of patients with chronic diseases in spiritual and secular forms of practice: Results with the shortened SpREUK-P SF17 questionnaire. *Integrative Medicine: A Clinician’s Journal, 11*(1), 28–38.

Carver, C. S. (1997). You want to measure coping but your protocol’s too long: Consider the brief COPE. *International Journal of Behavioral Medicine, 4*(1), 92–100. <https://doi.org/10.1207/s15327558ijbm0401_6>

Carver, C. S., Scheier, M. F., & Weintraub, J. K. (1989). Assessing coping strategies: A theoretically based approach. *Journal of Personality and Social Psychology, 56*(2), 267–283. [https://doi.org/10.1037/0022-3514.56.2.267](https://psycnet.apa.org/doi/10.1037/0022-3514.56.2.267)

Cloninger, C. R. (1994). *The Temperament and Character Inventory (TCI): A guide to its development and use*. Washington University, Center for Psychobiology of Personality.

Fuchshuber, J., & Unterrainer, H. F. (2021). “Test your spirituality in one minute or less”. Structural validity of the Multidimensional Inventory for Religious/Spiritual Well-being Short Version (MI-RSWB 12). *Frontiers in Psychology, 12*, Article 597565. <https://doi.org/10.3389/fpsyg.2021.597565>

Goebel, S., Mederer, D., & Mehdorn, H. M. (2018). Surgery-related coping in patients with intracranial tumors. *World Neurosurgery, 116*, e775–e782. <https://doi.org/10.1016/j.wneu.2018.05.091>

Grünke, B., Philipp, R., Vehling, S., Scheffold, K., Härter, M., Oechsle, K., et al. (2018). Measuring the psychological dimensions of quality of life in patients with advanced cancer: Psychometrics of the German Quality of Life at the End of Life-Cancer-Questionnaire. *Journal of Pain and Symptom Management, 55*(3), 985–991. <https://doi.org/10.1016/j.jpainsymman.2017.11.006>

Harzer, C. (2016). *WAMI (Work and meaning Inventory) German Version*. Greifswald University, Chair of Differential Psychology, Personality Psychology and Psychological Diagnostics.

Heuft, G. (2016). *Not lehrt (nicht) beten. Repräsentative Studie zu religiösen Einstellungen in der Allgemeinbevölkerung und von Patienten der psychosomatisch-psychotherapeutischen Ambulanz eines Universitätsklinikums.* Aschendorff.

Hood, R. W. (1975). The construction and preliminary validation of a measure of reported mystical experience. *Journal for the Scientific Study of Religion, 14*(1), 29–41.

Huber, S. (2003). *Zentralität und Inhalt: Ein neues multidimensionales Messmodell der Religiosität*. Leske und Budrich.

Huber, S. (2004). Zentralität und Inhalt. Eine Synthese der Messmodelle von Allport und Glock. In C. Zwingmann, & H. Moosbrugger (Eds.), *Messverfahren und Studien zu Gesundheit und Lebensbewältigung. Neue Beiträge zur Religionspsychologie* (pp. 79–105). Waxmann.

Hütter, B.-O., Huffmann, B., & Gilsbach, J.-M. (2020). Coping and health-related quality of life after closed head injury. *Clinical Neurology and Neurosurgery, 197*, Article 106194. <https://doi.org/10.1016/j.clineuro.2020.106194>

Knoll, N., Rieckmann, N., & Schwarzer, R. (2005). Coping as a mediator between personality and stress outcomes: A longitudinal study with cataract surgery patients. *European Journal of Personality, 19*(3), 229–247. <https://doi.org/10.1002/per.546>

Krohne, H. W., Bruin, J. T. de, El-Giamal, M., & Schmukle, S. C. (2000). The assessment of surgery-related coping: The Coping with Surgical Stress Scale (COSS). *Psychology & Health, 15*(1), 135–149. <https://doi.org/10.1080/08870440008400294>

Listopad, I. W., Esch, T., & Michaelsen, M. M. (2021). An empirical investigation of the relationship between spirituality, work culture, and burnout: The need for an extended health and disease model. *Frontiers in Psychology, 12*, Article 723884. <https://doi.org/10.3389/fpsyg.2021.723884>

Littmann-Ovadia, H. (2015). Brief report: Short form of the VIA Inventory of Strengths – construction and initial tests of reliability and validity. *International Journal of Humanities Social Sciences and Education, 2*(4), 229–237.

Lynn, B. T., Naughton, M. J., & VanderVeen, S. (2013). Faith at Work Scale. In J. Neal (Ed.), *Handbook of faith and spirituality at the workplace: Emerging research and practice* (pp. 419–427). Springer. <https://doi.org/10.1007/978-1-4614-5233-1_25>

MacDonald, D. A. (2000). Spirituality: Description, measurement and relation to the five factor model of personality. *Journal of Personality, 63*(1), 153–197. <https://doi.org/10.1111/1467-6494.t01-1-00094>

Moll, T. (2020). German-language scales for spirituality at work. *Journal of Management, Spirituality & Religion, 17*(3), 270–291. <https://doi.org/10.1080/14766086.2020.1765191>

Muthny, F. A. (1989). *Freiburger Fragebogen zur Krankheitsverarbeitung. Manual*. Beltz.

Norenzayan, A., & Hansen, I. G. (2006). Belief in supernatural agents in the face of death. *Personality and Social Psychology Bulletin, 32*(2), 174–187. <https://doi.org/10.1177/0146167205280251>

Paloutzian, R., & Ellison, C. W. (1982). Loneliness, spiritual well-being and the quality of life. In Peplau, L. A., & Perlman, D. (Eds.), *Loneliness. A sourcebook of current theory, research and therapy* (pp. 224–236). Wiley.

Peterman, A. H., Fitchett, G. Brady, M. J., Hernandez, L., & Cella, D. F. (2002). Measuring spiritual well-being in people with cancer: The Functional Assessment of Chronic Illness Therapy – Spiritual Well-Being Scale (FACIT-Sp). *Annals of Behavioral Medicine, 24*(1), 49–58. <https://doi.org/10.1207/S15324796ABM2401_06>

Peterson, C., & Seligman, M. E. P. (2004). *Character strength and virtues: A classification and handbook*. Oxford University Press.

Philipp, R., Vehling, S., Scheffold, K., Grünke, B., Härter, M., Mehnert, A., et al. (2017). Attachment insecurity in advanced cancer patients: Psychometric properties in the German version of the Brief Experiences in Close Relationships Scale (ECR-M16-G). *Journal of Pain and Symptom Management, 54*(4), 555–562. <https://doi.org/10.1016/j.jpainsymman.2017.07.026>

Rosenstiel, A., & Keefe, F. J. (1983). The use of coping strategies in chronic low back pain patients: Relationship to patient characteristics and current adjustment. *Pain, 17*(1), 33–44.

Ruch, W., Proyer, E. T., Harzer, C., Park, N., Peterson, C., & Seligman, M. E. P. (2010). Values in Action Inventory of Strengths (VIA-IS): Adaptation and validation of the German version and the development of a peer-rating form. *Journal of Individual Differences, 31*(3), 138–149. [https://doi.org/10.1027/1614-0001/a000022](https://psycnet.apa.org/doi/10.1027/1614-0001/a000022)

Satow, L. (2012). *SCI – Stress- und Copinginventar*. Retrieved March 29, 2025, from: <https://doi.org/10.23668/PSYCHARCHIVES.424>

Scheffold, K., Philipp, R., Vehling, S., Koranyi, S., Engelmann, D., Schulz-Kindermann, F., et al. (2019). Spiritual well-being mediates the association between attachment insecurity and psychological distress in advanced cancer patients. *Supportive Care in Cancer, 27*, 4317–4325. <https://doi.org/10.1007/s00520-019-04744-x>

Schnell, T., & Becker, P. (2007). *Fragebogen zu Lebensbedeutungen und Lebensinn: LEBE.* Hogrefe.

Steger, M. F., Dik, B. J., & Duffy, R. D. (2012). Measuring meaningful work: The Work as Meaning Inventory (WAMI). *Journal of Career Assessment, 20*(3), 322–337. <https://doi.org/10.1177/1069072711436160>

Streib, H., Klein, C., Keller, B., & Hood, R. (2021). The Mysticism Scale as a measure of subjective spirituality: New results with Hood’s M-Scale and the development of a short form. In A. L. Ai, P. Wink, R. F. Paloutzian, & K. A. Harris (Eds.), *Assessing spirituality in a diverse world* (pp. 467–491). Springer. <https://doi.org/10.1007/978-3-030-52140-0_19>

Unterrainer, H.-F., Huber, H.-P., Ladenhauf, K. H., Wallner-Liebmann, S. J., & Liebmann, P. M. (2010). MI-RSB 48 – Die Entwicklung eines multidimensionalen Inventars zum religiös-spirituellen Befinden. *Diagnostica, 56*(2), 82–93. <https://doi.org/10.1026/0012-1924/a000001>

Verra, M. L., Angst, F., Lehmann, S., & Aeschlimann, A. (2006). Translation, cross-cultural adaption, reliability, and validity of the German version of the Coping Strategies Questionnaire (CSQ-D). *Journal of Pain, 7*(5), 327–336. <https://doi.org/10.1016/j.jpin.2005.12.005>
